# Testing the effects of footwear on biomechanics of human body: A review

**DOI:** 10.1016/j.heliyon.2025.e42870

**Published:** 2025-02-20

**Authors:** Mohammad Mahdi Mohammadi, Amir Nourani

**Affiliations:** Department of Mechanical Engineering, Sharif University of Technology, Tehran, Iran

**Keywords:** Shoe sole, Sole mechanical tests, Shoe biomechanics, Sole structures, Sole types, Shoe pressure distribution, Subject specified shoes

## Abstract

Studies show the high potential of shoes to impact human movement and reduce the risk of injuries during normal and high-demanding activities. This review will delve into the existing literature on mechanical and biomechanical tests of shoes and their effects on the human body. Mechanical tests mainly include compression, bending, torsional flexibility, and impact tests. Biomechanical tests, on the other hand, study the kinematics and kinetics of the human body while performing different tasks. The primary goal of this review is to highlight the importance of isolating parameters in shoe design and testing to achieve optimal results in providing comfort, support, and injury prevention. Key conclusions include the influence of lattice structures on shoe stiffness and stress distribution, the effectiveness of composite loofah sponge for vibration damping, the benefits of Poron insoles for impact attenuation, the potential injury risk reduction with auxetic shoes, and the need for future research on mechanical tests, parameter investigation, and optimization of shoe sole structures.

## 1 Introduction

Shoes have been a vital part of our daily life since the prehistory era. Early shoes were made of natural materials namely leather, canvas, and wood. Throughout history, shoes encountered considerable changes in style, complexity, and material. The primary reason for these changes was to improve their performance in protecting our feet from harsh surfaces, providing more support and comfort, and also improving our movement style [[1], [2], [3]]. In consequence, with the increasing demand for engineered and specialized footwear, there has been a growing concern about evaluating the mechanical performance of shoes. As a result, mechanical experiments on shoes have been conducted for various purposes, including product development, quality control, and performance optimization. In addition, these experiments have also been used to investigate the effects of aging, wear and tear, and environmental factors on shoe performance. More importantly, the mechanical experiments of shoes are crucial in determining their effectiveness in protecting the feet, reducing fatigue, and improving athletic performance [4,5]. These factors can be evaluated through various tests, including compression, impact, torsion, and bending tests. These tests are conducted using specialized equipment that simulates real-world scenarios, such as walking, running, jumping, and landing. The results of these tests can provide valuable information about the strength, flexibility, and shock absorption properties of shoes [6,7].

A typical sports shoe features a tri-layered sole design comprising the insole, midsole, and outsole. The insole, positioned as the upper part of the shoe, is commonly constructed from lightweight nylon accompanied by a thin layer of rubber. Functioning as a buffer between the insole and outsole, the midsole serves to offer cushioning and regulate protonation. Meanwhile, the outsole, located at the base of the shoe, is responsible for creating traction against ground surfaces. It is typically fashioned from a slim, durable material known with high abrasion resistance, allowing it to provide both traction and flexibility, particularly in the forefoot area [8,9].

Moreover, early research shows that shoes can be a powerful tool to influence human movement and thus prevent injuries [[10], [11], [12]]. It seems that one of the causes of foot and back injuries is heavy physical movements and a proper shoe design is one possible way to protect people from them by optimizing movement kinematics and dynamics [4,13,14]. Thus, developing a proper design requires a full understanding of not only the mechanical properties of shoes but also the temporary or permanent effects of shoes on the human body during normal and heavy activities. For these reasons, lots of experiments have been conducted in recent years which can be divided into two major subjects namely (1) the mechanical properties and behavior of shoes, (2) the effect of footwear on kinematics, and the dynamic of the human body. To fully cover these two topics, the paper is structured as follows. First, the definition of shoe components is presented. Second, the multiple experiments and their reported results on the mechanical properties of the shoes will be discussed. Then, the effects of footwear on kinematics/kinetics of the human body will be reviewed. In the final section, the conclusion and the outlook of the paper will be reported.

Over the past 20 years, footwear science research has significantly grown, with many studies examining the impact of footwear on human movement. However, these studies often struggle to pinpoint which specific factors—such as material properties, design, or structure—are primarily responsible for the observed effects [15]. This study aims to address this issue by pursuing the following objectives:

1. Mechanical testing insights: Provide insights into selecting relevant mechanical tests to isolate key parameters for footwear, reducing costs and ensuring control prior to biomechanical testing.
2. Guidelines for shoe selection: Address the lack of practical guidelines in current literature by recommending shoes based on activities and physical needs to enhance efficiency and reduce the injury risk.
3. Modern sole structures: Introduce and highlight modern sole structures, such as lattice and auxetic designs, emphasizing their benefits like improved shock absorption and support.
4. Insole, midsole, and outsole structures: Introduce different structures and materials used in insole, midsole, and outsole components of footwear and their applications.

## 2 Research method

In this review article, the authors began by detailing the methodology for conducting conventional mechanical tests, including compression, bending, impact, and torsion tests, to assess footwear properties. They referenced previous studies that utilized these tests to evaluate footwear characteristics, providing examples from the literature. Subsequently, the authors explored the impact of different footwear types on body kinematics and kinetics during shoes common usage activities, such as studying the effects of weightlifting shoes on biomechanics during the barbell back squat exercise.

## 3 Mechanical tests

### 3.1 Compression test

Compression tests are used to compare different structures and materials for insoles and midsoles. The pressure on the sole can reach several times the body weight in athletes when jumping, and as a result, it can cause many injuries, including knee, ankle, and spine diseases. Optimizing the sole's characteristics can help reduce these risks. In this section, after reviewing efforts to standardize pressure tests, the impact of changes in the structure and materials of insoles and midsoles on performance in compression tests will be discussed.

**Fig. 1:**
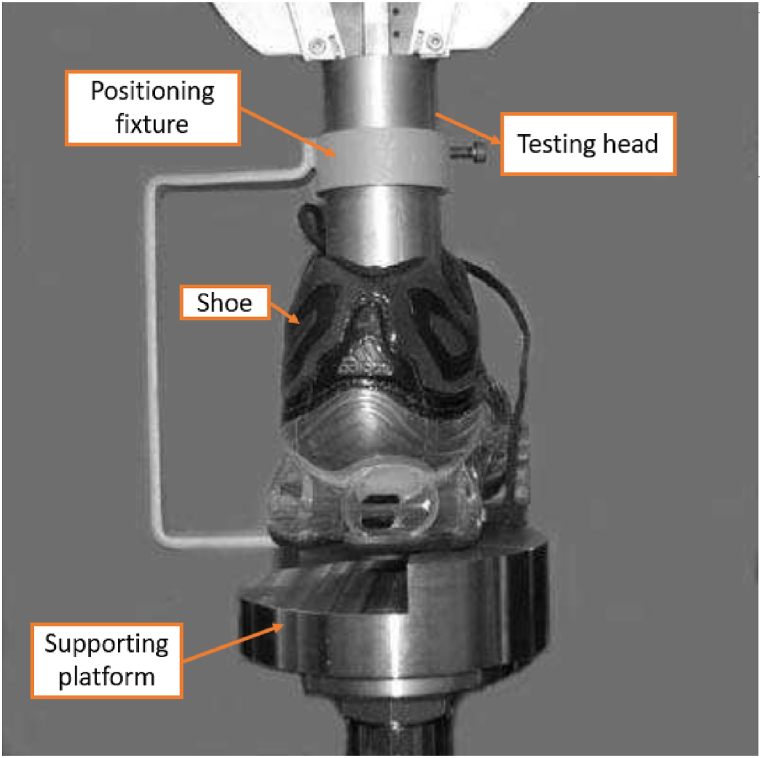
Dickson et al. [15] testing device.

Few attempts have been made to standardize shoe pressure testing. Dickson et al. [15] conducted tests on different heel segments of two commercially available running shoes (Mizuno and Adidas shoes) to develop a standardized test method for characterizing the stiffness of heel shoe segments of sports shoes. The experimentation involved the utilization of an Instron 5569 equipped with a 50 kN load cell to conduct assessments. Pressure was applied to the innersole, specifically targeting the heel area. For the complete heel test, a flat anvil was employed to secure the shoe entirely. Conversely, in the segmented test, an anvil was positioned only on a section of the heel (Fig. 1). The results showed the necessity of executing multiple complete heel compressions to attain a stable testing state. Due to their results, the Adidas shoe (Ambition PB) showed less stiffness than the Mizuno shoe (Alchemy) in the whole heel test particularly evident through a notable stiffness surge in the rearfoot at a compression level of 3 mm. Notably, the Mizuno shoe demonstrated superior energy absorption owing to its foam-based Mizuno sole, as outlined in the data provided in Table 1. However, other factors such as sole geometry and structure were not isolated in the analysis.

**Table 1:** Dickson et al. [15] energy absorption.

Portion	Average energy absorption %	St. Dev. %
Adidas Whole Heel	25.96	1.42
Adidas Lateral Heel	24.61	1.89
Adidas Medial Heel	21.96	1.15
Adidas Posterior Heel	21.93	1.64
Mizuno Whole Heel	32.61	2.22
Mizuno Lateral Heel	36.96	1.98
Mizuno Medial Heel	34.98	0.99
Mizuno Posterior Heel	32.58	1.95

#### 3.1.1 Midsole compression tests

**Fig. 2:**
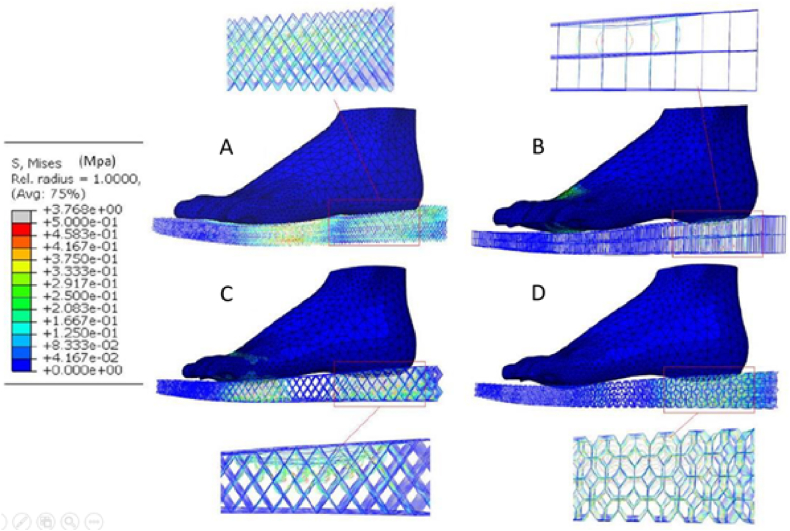
Dong et al. [17] topologies and their stress distributions: (A) Diamond, (B) Grid, (C) X shape, and (D) Vintiles.

**Fig. 3:**
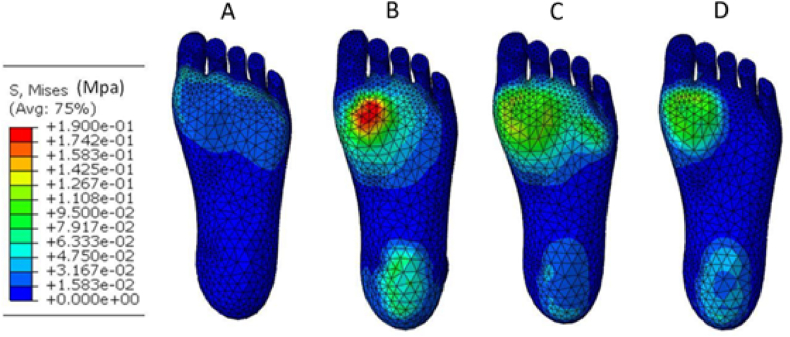
Dong et al. [17] plantar stress distributions: (A) Diamond, (B) Grid, (C) X shape, and (D) Vintiles.

**Fig. 4:**
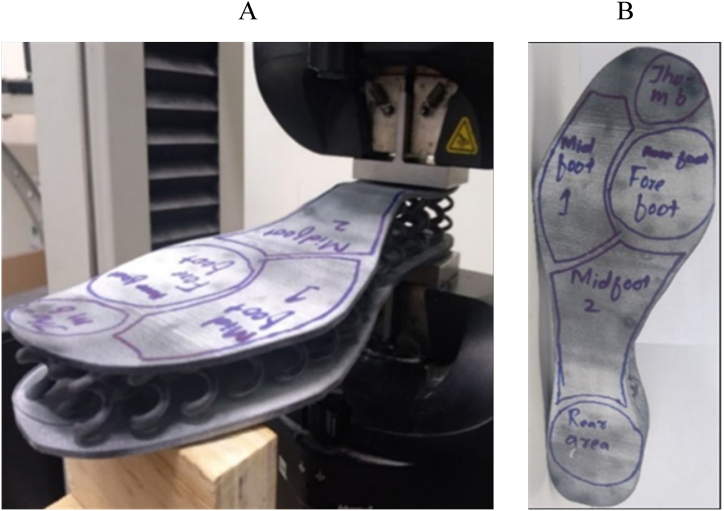
Uniaxial compression loading-unloading test of additively manufactured midsoles (A) for five different zones (B) [16].

An important factor that can influence the stiffness of shoe soles and may change the athletic performance is the midsole structure. Using finite element (FE), the mechanical response of lattice-designed shoe soles (Fig. 2) under the compression caused by the human foot was examined numerically. Experiments were also conducted to explore discrepancies among the mechanical behaviors of the four lattice shoe soles. Observations of shoe sole deformation indicated distinct characteristics across topologies: the Grid topology exhibited buckling, while other topologies showcased substantial bending. Among these, the X shape demonstrated initial densification, reaching a 2000 N load after a 10 mm displacement, followed by Vintiles, whereas Diamond exhibited densification last. Simulation results indicated that the Grid lattice structure yielded the highest stiffness among the designs, while the Diamond lattice structure ranked as the least robust (Fig. 3). Mubasher et al. [16] designed two shoe midsoles using variable-dimension helical springs (VDS) and their performance was compared with a third shoe midsole designed using uniform-dimension helical springs (UDS). Spring dimension and mass were adjusted according to each zone height and plantar force distribution data. Experimental boundary conditions were applied to the testing specimen as seen in Fig. 4. The UDS midsole exhibited a significantly higher distortion (45 %) compared to the VDS midsole (24 %) during the loading-unloading experiment. The results have proven that the midsole design with the VDS structure could achieve higher stability (i.e., the consistent behavior and minimal fluctuation in material setting or deformation of midsoles during repeated loading-unloading iterations) and a larger strength-to-weight ratio with a flexible behavior. These studies suggest research that did not isolate sole structure may lack full validity. It also recommends that shoe manufacturers consider incorporating a variety of sole designs to offer customers a broader range of stiffness tailored to their needs and common activities. For instance, high-demand activities may benefit from soles with greater stiffness, even if this comes at a higher cost.

**Fig. 5:**
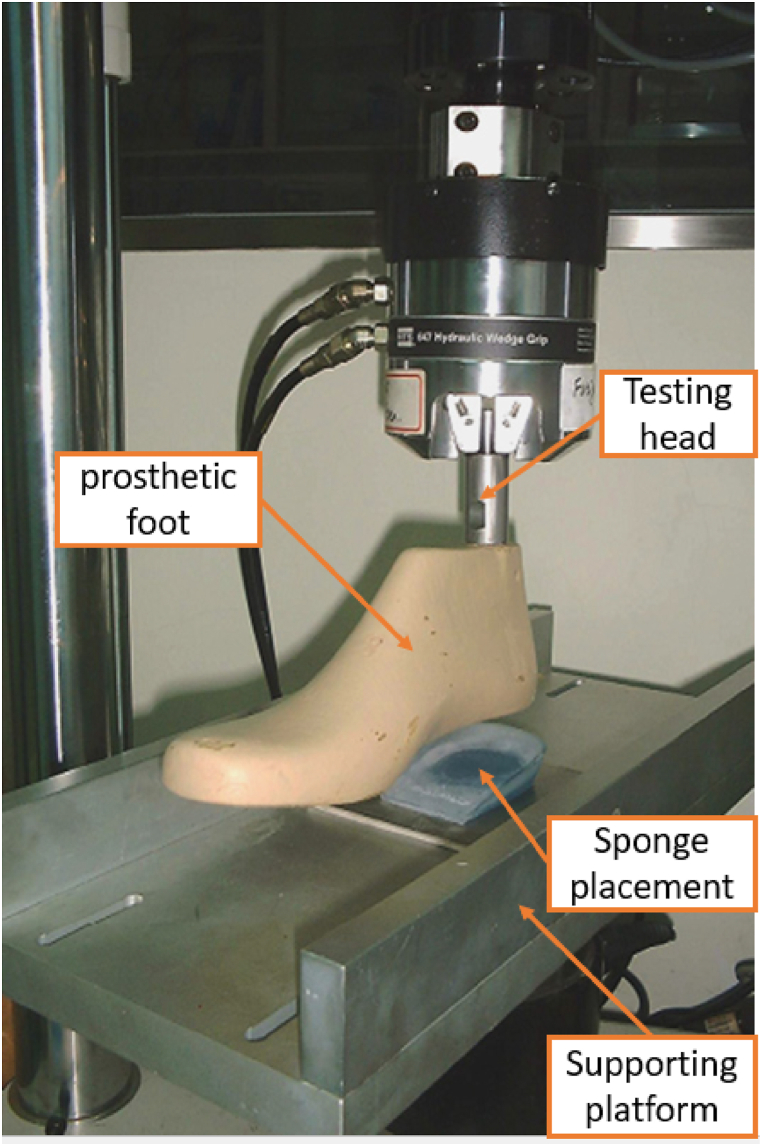
Akano et al. experimental setup [20].

**Fig. 6:**
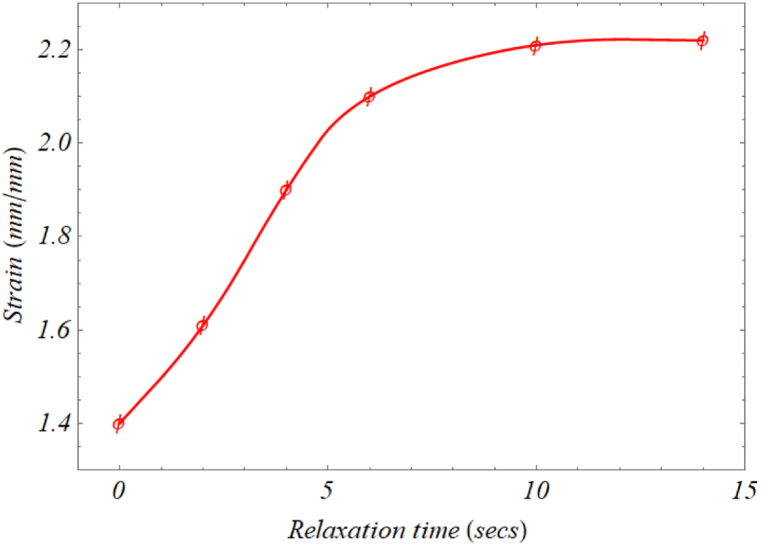
Compression analysis test curve [20].

Another key factor that can optimize shoe stiffness and help prevent injuries during high-demand activities is the choice of sole material [18]. Bruckner et al. [19] conducted long-term (240,000 load cycles) and short-term (100 load cycles) compression tests on running shoes with midsoles made of 7 polyurethane (PU) and 2 ethyl vinyl acetate (EVA) materials. They used a servo-hydraulic testing device with a spherical-shaped stamp (50 mm diameter) and found that PU foams with special characteristics are suitable as the midsole material due to their damping properties. Akano and Suberu [20] compressed the sample of composite loofah sponge using an 80 kg compressive force applied for about 2.5 h with an ASTM E606 machine (Fig. 5). They measured and recorded the height of the sponge before releasing the load. After releasing the load, the sponge's height was recorded at 2-s intervals until it returned to its original height, as shown in Fig. 6. The researchers also conducted strain-controlled low and high cycle fatigue testing, measuring and recording the low and high strain rates, as well as the relaxation time. The key characteristic of the composite loofah sponge was its ability to effectively dampen vibrations, resulting in minimal impact on the knees and ankles.

#### 3.1.2 Insole compression tests

Another factor that studies should consider its isolated effect is the insole material/structure. Sun et al. [21] conducted an axial compression test to compare two insole materials (silicone and thermoplastic elastomer (TPE)). They conducted the study involving two materials characterized by three hardness values each (Shore-C 15°, 20°, and 25°), and varying thicknesses spanning 6, 8, 10, 12, 14, and 16 mm. The materials were securely positioned and subjected to compression using a predetermined loading-unloading rate (525 and −525 N/s), mimicking the forces experienced during actual walking steps. The simulation aimed to replicate a peak impact of approximately 1.5 times the body weight of an individual weighing around 70 kg. The force was fluctuated between 0 and 1050 N using a load control mode. Notably, after 20 cycles, a considerably more substantial decrease in the hysteresis loop was observed in the silicone material compared to the TPE.

**Fig. 7:**
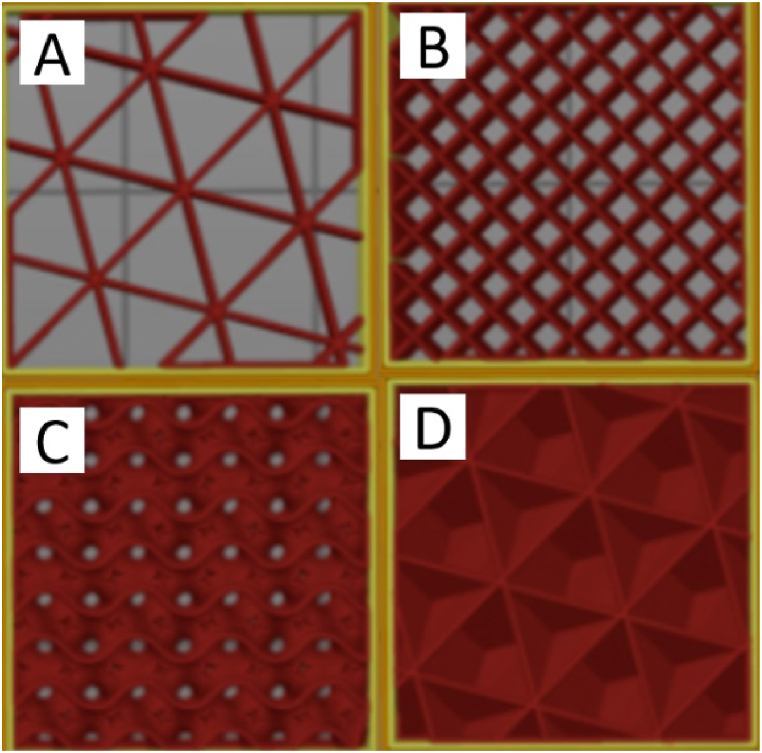
Orsu and Shaik patterns: A) Triangle B) Rectilinear C) Gyroid D) Cubic [23].

Orsu and Shaik [22] proposed a method to increase the efficiency of shoe insoles in various usages tailored by 3D printing. This study used PolyFlex thermoplastic polyurethane (TPU) 90 A, because of its suitable properties to produce the shoe insole. In this study, the design of the insole was taken from the “Gensole Software”. Compression tests were done utilizing a universal testing machine (UTM). Higher infill percentages, specifically 60 %, 40 %, and 25 % were tested with different patterns (Fig. 7); triangle (Fig. 7-A) and rectilinear (Fig. 7-B) patterns showed better compression modulus against weight ratio at 60 % infill, gyroid (Fig. 7-C), and triangle patterns at 40 % infill, and gyroid and triangle patterns again at 25 % infill. However, when considering the ratio of compression modulus against strain, they found that at 60 % infill, rectilinear and triangle patterns experience a higher ratio than others (Fig. 7-C and D), at 40 % infill triangle and rectilinear and at 25 % infill triangle, gyroid, and rectilinear. Two previous studies indicate that using different insoles can change the stiffness and damping properties of shoes, potentially affecting the interpretation of results reported in shoe studies.

### 3.2 Bending tests

The bending test – as one of the practical examinations for shoes-can be broadly divided into a simple test and a three-point bending test. The general process of conducting a sole bending test is as follows:

1 Assessment of Bending Stiffness:
• Measure the bending stiffness of various shoe soles using appropriate testing equipment.
• Calculate the maximum angle the sole reaches corresponding to these bending stiffness values.
2 Comparison with Human Test Data:
• Gather test data from individuals engaged in the desired activity (e.g., walking, standing, jumping).
• Record the desired kinematic and kinetic parameters such as Lumbosacral disc compression force and lower limb kinematics.
3. Analysis and Decision-Making:
• Compare the calculated maximum angles of sole bending stiffness with the human test data to determine correlation and suitability.
• Consider other factors like durability, support, and comfort alongside bending stiffness for comprehensive decision-making.

#### 3.2.1 Simple bending test

**Fig. 8:**
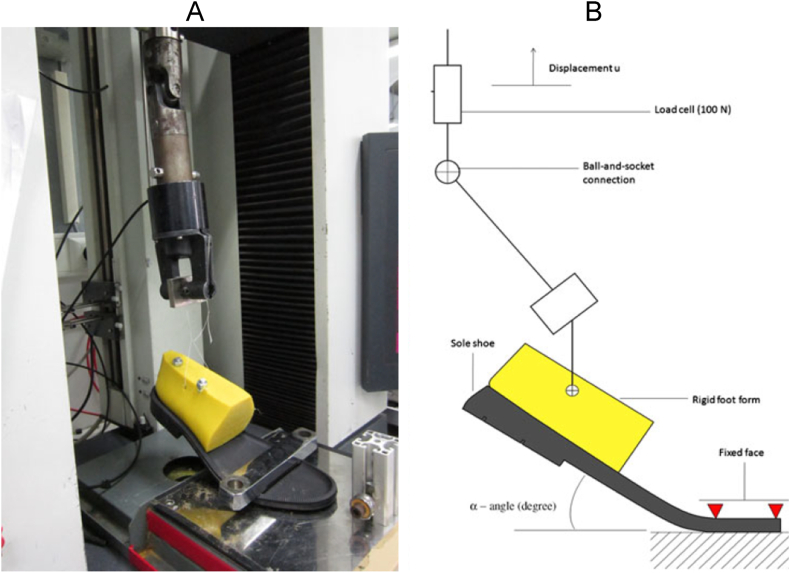
(A) Bending test practical application and (B) its scheme [24].

In this test, the forefoot is fixed and the back part is pulled by a strong wire (Fig. 8-A). The schematic is shown in Fig. 8-B.

**Fig. 9:**
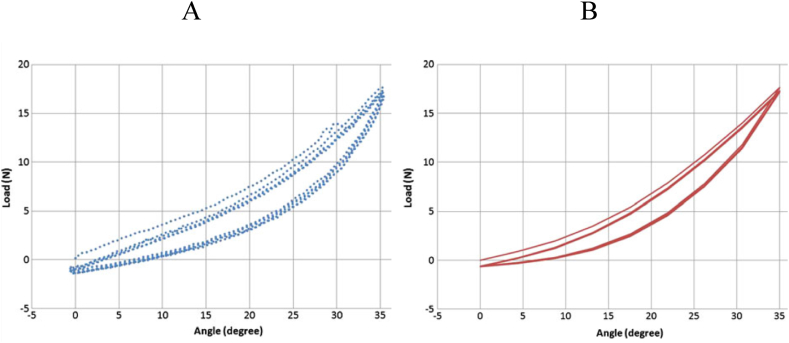
Experimental (A) and Numerical (B) simulated hysteresis curves [24].

Benkahla et al. [24] conducted a simple bending test on an NBR (Nitrile-Butadiene-Rubber) outsole. The experimental procedure involved securing the front part of the outsole, while the back and heel were affixed to a rigid section of a foot form made of polyethylene. Using an MTS tensile machine, vertical displacement was applied, allowing movement of the rigid part via a ball-and-socket connection, initiating a bending motion in the sole. The vertical displacement rate was set at 540 mm/min, and load measurements were taken. Subsequently, cyclic loading mimicking a triangular signal with a maximum angle amplitude of 35° was introduced. Additionally, numerical simulations were conducted using Code-Aster with a visco-hyperelastic constitutive model incorporating discontinuous damage to simulate the Mullins effect. Fig. 9 illustrates the hysteresis curve responses for five bending cycles, displaying both the experimental and computed data. Notably, a high level of agreement was observed between the experimental results and the outcomes derived from numerical simulations. However, this protocol and constitutive model are tailored to the NBR material and a specific outsole structure, and they may not yield accurate results for other sole materials or outsole designs with varying thicknesses.

Nunns et al. [25] conducted a simple bending test to show that creating different structures on the outsole can be effective in improving the bending properties of shoes. They used two boot models (8 and 6 cleats) and assessed the boot outsole stiffness by measuring the peak force required to bend the outsole of each boot at 45°. The required mean peak force to bend the outsole was 42.4 N for the eight-cleat arrangement and 59.6 N for the boot six-cleat arrangement. However, the study has some limitations; testing the outsole and insole separately may overlook how they interact together in the complete footwear system, which can affect comfort and performance. Additionally, the impact test used a fixed mass and velocity, which may not capture the wide range of forces experienced in real-life activities. This could limit the applicability of the results to dynamic real-world conditions.

In addition to specific limitations noted earlier, the studies discussed in this section also have several common shortcomings. These include small sample sizes - that may affect the applicability of findings - and controlled testing environments that might not accurately reflect real-world conditions. Additionally, a focus on short-term results may overlook the long-term effects on performance and injury risk. Future research should investigate the long-term impacts of different designs while considering the specific needs of athletes and environmental factors, such as humidity and temperature, in various sports.

#### 3.2.2 Three-point bending test

**Fig. 10:**
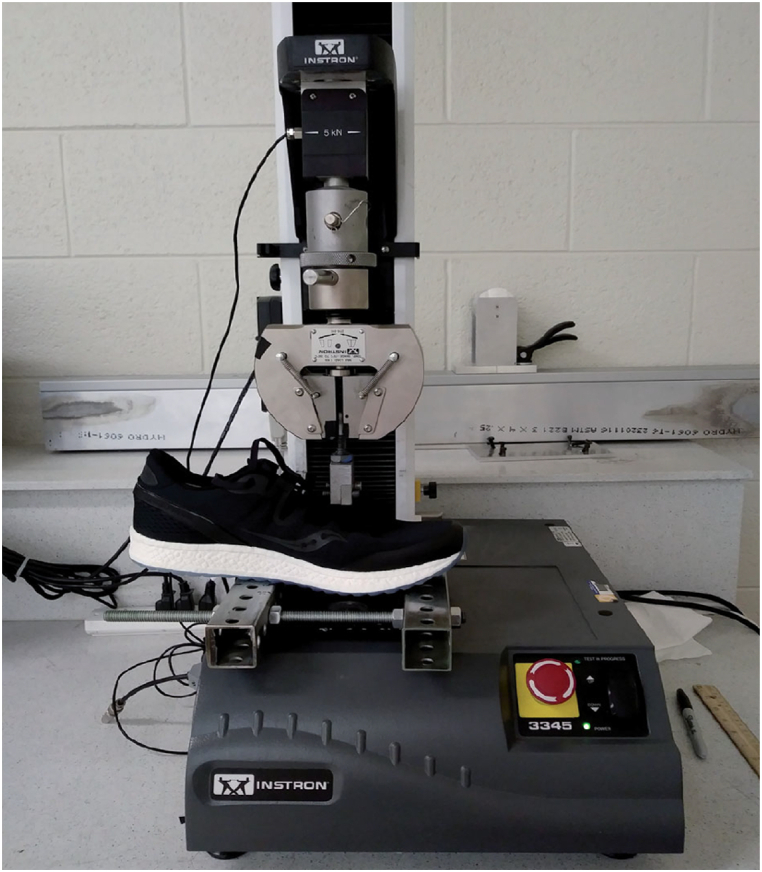
A schematic of a 3-point bending test [28].

Three-point bending is the most popular bending test in shoe biomechanics. In this type of test, the forefoot and rearfoot are fixed and the sole is stretched vertically from the midfoot part (Fig. 10).

One way to improve running economy is by increasing bending stiffness of shoes [26]. Ortega et al. [27] reviewed lots of three-point bending tests and collected their bending stiffnesses to help researchers choose the best shoes for their running economy. Their conclusion highlighted that the existing body of literature indicates alterations in running economy linked to increased longitudinal bending stiffness, spanning from around a 3 % decrease to a 3 % increase.

### 3.3 Torsional flexibility test

**Fig. 11:**
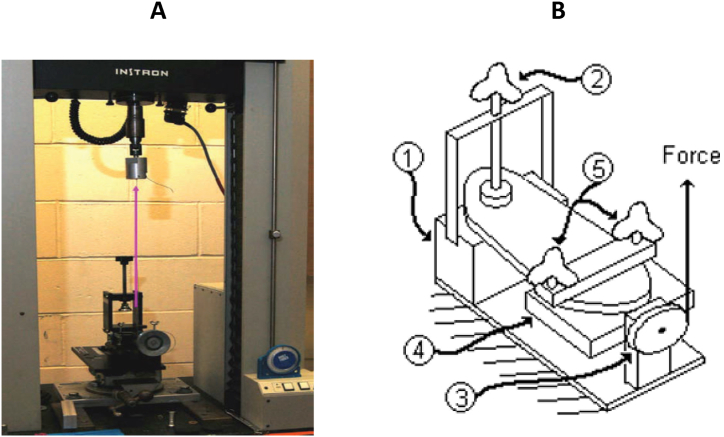
(A) Structural testing machine [29]. (B) Torsional flexibility setup components [31]: 1- Rearfoot fixing plate. 2- Rearfoot fixing knob 3- Pully 4- Forefoot plate 5- Forefoot knobs.

**Fig. 12:**
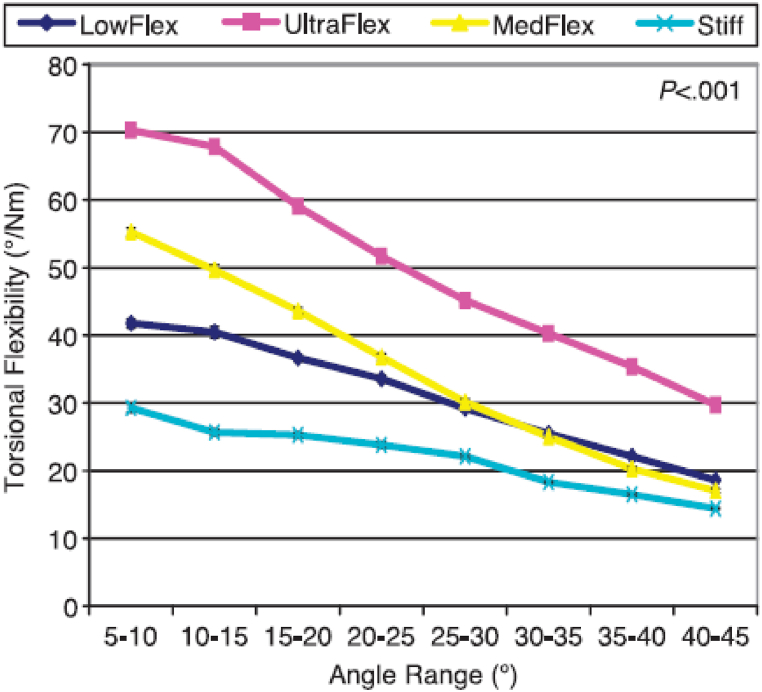
The torsional flexibility of different shoes depicted in response to applied moment, represented by the slope of the moment-angle curve.

Torsional flexibility refers to the ability of a structure or material to resist or accommodate twisting forces or torques applied to it. Limited studies have investigated the torsional flexibility testing of shoes. Buckland et al. [29,30] have conducted some of the most prominent studies using this type of test. They limited their studies to new walker children and in two separate studies they evaluated the pressure distribution and body stability of these subjects while wearing shoes with different torsional stiffnesses (UltraFlex, MidFlex, LowFlex, and Stiff) [29,30]. To evaluate torsional flexibility in various shoe designs, they utilized a structural testing machine (Instron 4201 tabletop, Norwood, Massachusetts) along with a customized jig. This jig converted tensile loading into a torsional moment (Fig. 11). The testing procedure involved securing the rearfoot while the forefoot was firmly held in place by a clamping apparatus, allowing free rotation about the shoe's longitudinal axis. Increasing the cross-head speed of the head and load cell caused tension in the cable depicted in Fig. 11 - A, subsequently rotating the pulley connected to the toebox (Fig. 11- B). They observed a consistent decrease in torsional flexibility across all shoes as the applied moment increased. In this study, it was found that the Stiff shoe resulted in the lowest peak pressures among the participants. Conversely, the UltraFlex shoe showed elevated peak plantar pressures (Fig. 12), often comparable to those experienced during barefoot loading across various regions of the foot. Overall, while these findings contribute valuable insights into the relationship between torsional flexibility and shoe performance, the limitations of a narrow range of shoe designs, materials, environments, and sample sizes call for further investigations. Specifically, exploring auxetic structures, such as the use of rotating triangles in the outsole or re-entrant geometries in the midsole, could potentially enhance torsional flexibility and reduce the risk of foot injuries.

### 3.4 Impact test

**Fig. 13:**
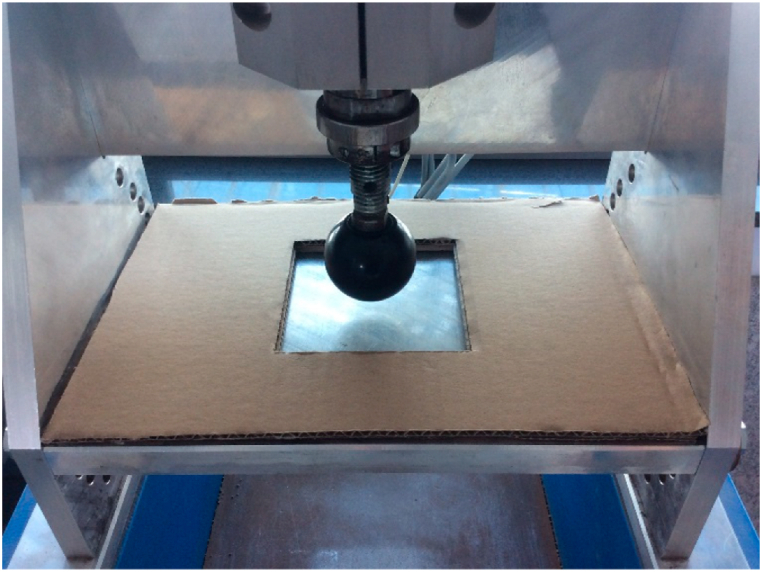
Keshvari et al. [33] impactor machine.

Shoe impact testing is an important test that measures a shoe potential to absorb impact and protect the wearer's foot from sudden shocks or accidents. Delattre and Cariou [32] focused on users' perception of footwear cushioning. They evaluated 31 commercially available running shoe models by subjecting the rearfoot to an impact test involving a 50 mm free fall of a 6.5 kg mass. Each shoe underwent 30 cycles, and the last five cycles were selected for analysis. Using a sensory-trained panel method, they quantified users' perception of shoe cushioning intensity. Their study revealed a notable correlation, enabling the prediction of users' perception of heel penetration based on the shoe's absolute maximal compression. In addition, Keshvari et al. [33] conducted a dynamic shock absorption test for the measurement of cushioning properties. They used the pneumatic impactor device (5 cm diameter and 4.3 kg mass) and allowed the weight to fall from a height of 6 mm (Fig. 13). They found that the insole with the lowest impact peak acceleration was rated as the most comfortable, except for ethylene-propylene-diene-monomer (EPDM) insole which had the highest impact peak acceleration but was still rated as the most comfortable with respect to cushioning. Based on findings of this study, optimizing a shoe's impact peak acceleration may satisfy a wide range of users by enhancing its compression properties. This highlights the need for further detailed research in this area to better meet diverse consumer needs and preferences. In another study [25], the impact attenuation properties of the Poron and Poron/gel insoles were measured. A missile (45 mm diameter and 8.5 kg mass) was set to impact rearfoot, with the insole positioned inside as typically worn during normal use, then it was released with a velocity of 92 cm s^−1^. They observed the Poron insole offered superior impact attenuation compared to the Poron/gel condition. However, although the latter did not evaluate the insoles in various environments or under different velocities and heights, it still supports the preference for Poron insoles over Poron gels for normal usages.

Few efforts have been made in order to simulate the interaction between shoe and foot dorsum. Rupérez et al. [34] proposed a linear elastic orthotropic model designed for simulating the deformation of upper shoe materials during the gait cycle. Their study involved conducting tests to evaluate leather resistance during lasting and to mimic the shoe-forming process using lasts. The examination of damage resistance adhered to ISO 17693:2004 standards and utilized the electronic elastometer model 5507-E provided by Muver S.L [35]. In the shoe-forming process using lasts, a cylindrical component composed of identical material to that of the shoe lasts applies pressure to the material sample. This experimental setup emulates the mechanism by which the shoe upper material is pressed by the foot dorsum during walking, considering the foot dorsum's minimal soft tissue presence. The findings from both tests demonstrated the applicability of an orthotropic model in accurately representing the behavior of calfskin material. However, the applicability of these findings may be restricted since the study concentrated mainly on calfskin and did not explore variations in loads associated with different gait patterns or activities. Despite this limitation, the importance of the shoe dorsum in overall comfort suggests that these models have significant potential to improve comfort for both the foot dorsum and the shoe sole.

## 4 Effects of shoes on the kinematics and kinetics of the human body

**Fig. 14:**
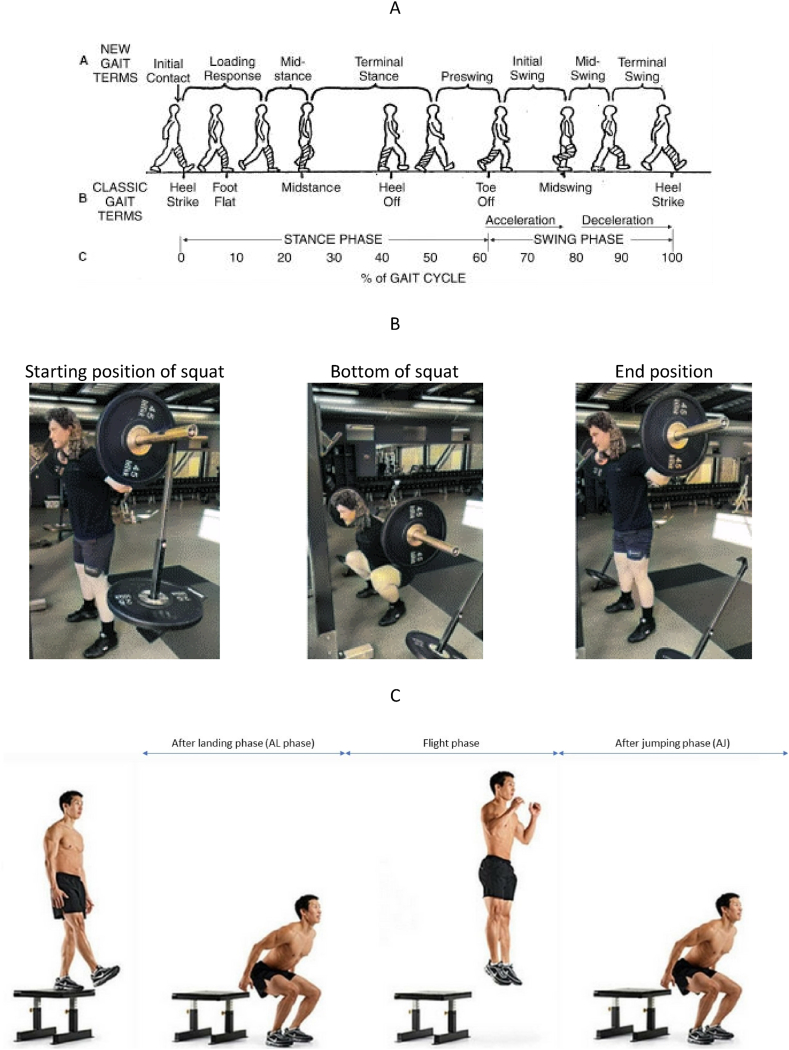
Activities investigated in this study: (A) Gait cycle [39]. (B) Barbell back squats [40]. (C) Drop vertical jump [37].

The kinematics and kinetics of the human body undergo alterations during various activities, and these changes can have both temporary and permanent effects on an individual's health. Selecting an appropriate footwear plays a crucial role in promoting proper alignment, stability, and overall performance while mitigating potential adverse effects on the body's biomechanics. Different types of shoes, each with unique features, have been examined in specific activities such as gait, standing posture, drop vertical jumps, and barbell back squats. Gait consists of two phases: the stance phase, when the leg contacts the ground, and the swing phase, when it is off the ground, optimizing energy consumption and kinematics (Fig. 14-A) [36]. Squats, which can be performed with or without a barbell, involve transitions through three main positions: the start, the bottom, and the end, and are beneficial for both rehabilitation and weightlifting (Fig. 14-B) [37]. The drop vertical jump involves the landing, flight, and takeoff phases and is commonly used in sports requiring frequent jumping and landing, such as basketball (Fig. 14-C) [38]. This review provides valuable insights into how different types of footwear impact specific movements and postures, offering a deeper understanding of how to optimize biomechanical efficiency tailored to each unique activity. Table 2 highlights the characteristics of footwear in their common activities. While some footwear may also be suitable for less typical activities, this table serves as a helpful guide for selecting the most appropriate option for your specific needs. A detailed analysis of the studied footwear types will be provided in the following section.

**Table 2:** Footwear applications in their common activities.

Common activity	Footwear	application
Gait	Forefoot lateral unstable shoes	strengthening the lower leg and improving ankle stability.
	Forefoot medial unstable shoes	enhancing both lower leg and thigh strength.
	Forefoot Neutral unstable shoes	focusing on lateral stability.
	Rear foot anterio-posterior unstable shoes	Boosting plantarflexion/dorsiflexion muscle activation
	Rearfoot medio-lateral unstable shoes	Enhancing ankle inversion/eversion muscle strength
	Off-loader shoes	Redistributing pressure for ulcer management; reducing forefoot load.
	High-heels	Increase a person's attractiveness by increasing lumbar lordosis.
	Specialized pregnancy footwear	improve comfort, spinal alignment, and hip flexion during pregnancy and postpartum recovery.
	Barefoot-inspired shoes	promotes a gait closer to the natural barefoot walking pattern
	Anti-pronation footwear	correcting excessive inward foot rolling by enhancing overall foot stability.
	Gender specific shoes	Improving knee abduction and ankle eversion in women, and hip flexion in men
	Running shoes	Improving gait by providing customers with a wide range of footwear options.
Squat	Weightlifting shoes	Improving weightlifting performance for novices and experienced lifters
Drop jump	Auxetic shoes	Reducing peak L5-S1 disc pressure to prevent low back injuries.
	Basketball shoes	Reducing foot injuries by choosing the optimum hardness; inadequate hardness may not benefit individuals.

### 4.1 Gait and standing posture

Numerous researchers have conducted studies to examine the impact of various shoe types on gait and standing activities. Human locomotion encompasses diverse gaits, such as walking, running, and jogging, appreciated for their energy-efficient nature [41].

#### 4.1.1 Unstable shoes

Wearing a rounded shoe sole design in the anterior-posterior direction makes the shoe unstable, consequently, influencing an individual's kinetics during gait and standing postures. The initial studies revealed that unstable shoes exhibited a significant increase in dorsiflexion of the ankle joint during the stance's initial phase, alongside a notable increase in the center of force excursion in both the anterior-posterior and mediolateral directions [42]. Prolonged wearing of these shoes may result in a reduction of anterior-posterior displacement and anterior-posterior TR (Trembling) (oscillatory movements around the reference point caused by spinal reflexes and changes in the mechanical properties of muscles and joints) RMS (Root mean square), while simultaneously increasing anterior-posterior RM (Rambling) (movement of a reference point for postural sway influenced by supraspinal processes) mean velocity and mediolateral RM displacement [43]. The study's outcomes emphasized that standing in unstable MBT footwear, as opposed to conventional flat control shoes, induced a more pronounced thoracic kyphosis and caused heightened mobility at the thoracolumbar and lumbopelvic levels. Concurrently, wearing unstable shoes amplified muscle activity in the TA (tibialis anterior), PL (peroneus longus), LG (lateral gastrocnemius), and lumbar erector spinae, while demonstrating no discernible impact on the activities of the abdominal, upper thoracic paraspinal, or lower thoracic erector spinal muscles [44,45]. Using unstable footwear also results in notably longer step lengths and a reduced cadence in comparison to conventional shoes, regardless of the walking or running speed [46]. The results of these studies demonstrate that prolonged wear of unstable shoes (e.g., over a period of 5 weeks) may alter muscle activation patterns, leading to changes in kinematics over time, ultimately improving individuals' stability.

**Fig. 15:**
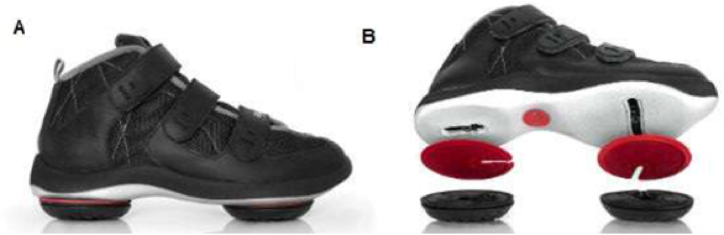
Biomechanical device: (A) Unstable with adjustable elements. (B) stable without any attached elements [47].

In 2019 a unique footworn device was developed. This device was known to turn into unstable shoes by connecting the hemisphere elements to the outsole (Fig. 15). In one particular investigation, comparisons were made between unstable shoes equipped with the mentioned device (Fig. 15-A) and simple shoes (Fig. 15-B). The findings revealed a substantial reduction in average hip flexion (10 % during the midstance phase), increased ankle dorsiflexion (56 % and 8 % during the midstance and terminal stance phases of the gait cycle, respectively), and inversion (21 % during the terminal stance phase). Moreover, there was an increase in mean spine extension (11 % during midstance) and contralateral flexion (43 % during terminal stance), alongside a decrease in mean internal spine rotation (67 % during midstance) while wearing unstable shoes. Additionally, the variability of spine internal rotation angle significantly increased (39 % during midstance), while the variability of shoulder adduction/abduction angle decreased (38 % and 49 % during midstance and terminal stance, respectively) [47]. The use of this device made it possible to compare different unstable shoes with instability in different regions of the shoe. For instance, variations such as medial unstable (MU, primarily supporting the first and second metatarsal bones), neutral unstable (NU, mainly supporting the middle metatarsals), and lateral unstable (LU, primarily supporting the lateral metatarsals) can be achieved by linking these components to the forefoot. Alterations in the positioning of these unstable elements in the forefoot distinctly impact knee kinematics. While NU exhibits minimal effects on knee kinematics, more noticeable changes are observed in LU and MU conditions. When comparing hip rotation angles, all three types of unstable shoes demonstrate a decrease in hip external rotation amplitude and an increase in hip internal rotation amplitude. Particularly, the data from LU and MU indicate no inclination for external rotation of the hip, differing notably from both simple and NU shoes. There are no discernible differences in hip flexion-extension and abduction-adduction between simple and unstable shoes. Furthermore, variations among different unstable shoes and simple shoes in ankle dorsiflexion are negligible, yet positional adjustments of unstable elements in the forefoot's coronal plane significantly impact ankle inversion, eversion, and rotation, particularly during the weight-bearing phase [45]. As a result of this study, athletes can use unstable shoes to target specific muscles during exercise. LU was found to significantly increase the activity of the tibialis anterior (TA), peroneus longus (PL), and lateral gastrocnemius (LG), making them ideal for strengthening the lower leg and improving ankle stability. MU boost the activity of the PL, LG, and rectus femoris (RF), enhancing both lower leg and thigh strength. NU primarily engage the PL, focusing on lateral stability. By selecting the right type of unstable shoe, athletes can more effectively target key muscles and improve their performances. Both anterio-posterior (AP) and medio-lateral (ML) instability can manifest in the rearfoot, with unstable shoes of both types reducing the moment and power on lower limb joints, thereby decreasing impact loads and increasing joint angles; however, these changes suggest that other biomechanical characteristics may also be affected [48,49]. Zhou et al. [50] indicate that footwear designed for anterio-posterior instability can boost the activation of muscles responsible for ankle plantarflexion and dorsiflexion, while shoes designed for medio-lateral instability may enhance muscle strength for ankle inversion and eversion, providing better protection for the lower limbs and closely resembling natural human movement. AP unstable shoes are designed to introduce a new way of walking by intentionally creating slight instability, which aims to improve balance and posture over time. Adults may find it easier to accept AP shoes because they are accustomed to the stability and support of modern footwear. In contrast, ML unstable shoes are even less stable, mimicking the experience of walking barefoot. Encouraging the use of ML unstable shoes from a young age can be beneficial for long-term physical development, as they increase lower limb muscle activity [50]. The level of instability can be heightened by adjusting the height of the instability elements within these devices. This adjustment can notably affect the magnitude of peak values in both hip extension and ankle inversion angles across different stages of instability [51]. Physicians may recommend specific levels of instability for athletes based on their individual physical characteristics.

**Fig. 16:**
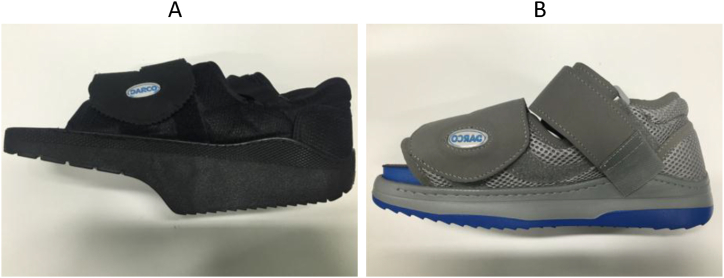
Off-loader shoes: (A) The right side is the new design (Relief Dual shoes) and (B) the left side is the old design (Ortho-Wedge shoes).

Off-loader shoe models, including the Ortho-Wedge and Relief Dual designs (Fig. 16), serve the purpose of alleviating pressure from specific foot areas by redistributing it to other regions. In a study by Michalik et al. [52], the effects of these shoes on gait cycle parameters and spine posture were explored. Their findings demonstrated significant changes in hip and spine positions during both standing and walking for both shoe designs. The Ortho-Wedge shoe, characterized by a high-profile midfoot and rearfoot outsole along with a low-profile forefoot sole, effectively shifted body weight towards the midfoot and rearfoot, consequently reducing pressure on the forefoot area. Conversely, the Relief Dual shoe featured a low-profile sole without a raised forefoot, employing multiple sole layers and a rigid shank to distribute weight. The study revealed that the older Ortho-Wedge shoes impacted gait parameters significantly more than the newer Relief Dual shoes, suggesting that the latter may encourage more natural movements than their predecessors. Physicians often recommend wearing a specialized twin shoe to mitigate the negative effects associated with these designs. Nevertheless, despite their drawbacks, these shoes are essential in managing Diabetic Foot Ulcers, as they effectively redistribute pressure away from ulcerated areas, promoting healing and preventing further injury.

While these studies provide valuable insights into unstable shoes, several concerns remain. It is crucial to consider gender when evaluating their therapeutic effects because men and women use different strategies for controlling ankle movement; thus, some effects may not be applicable to both genders. Additionally, varying hardness in shoes can significantly influence the movements of the ankle, hip, and pelvis, indicating that some effects of unstable footwear may stem from differences in stiffness. Furthermore, many studies fail to investigate the impacts of velocity and manufacturing processes on these shoes. Future research should aim to conduct mechanical tests to identify and isolate the most critical parameters that affect the effectiveness of unstable footwear. Another approach is using feature extraction in gait analysis, which helps researchers identify the key gait pattern characteristics altered by different shoes. This method allows for a more precise comparison of shoes based on their main gait-influencing features [53].

#### 4.1.2 High-heels

**Fig. 17:**
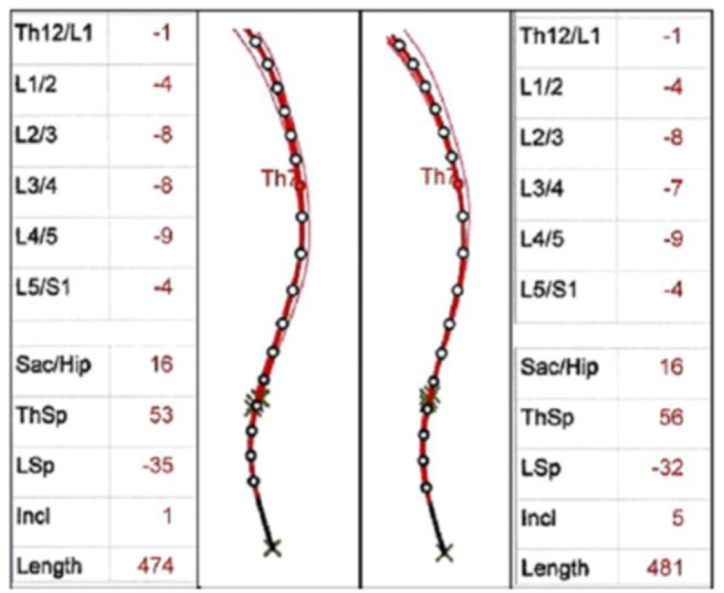
The shape of the spine measured by the spinal mouse in different discs. Left side before wearing high-heels and right side after wearing high-heels for 10 min [55].

**Fig. 18:**
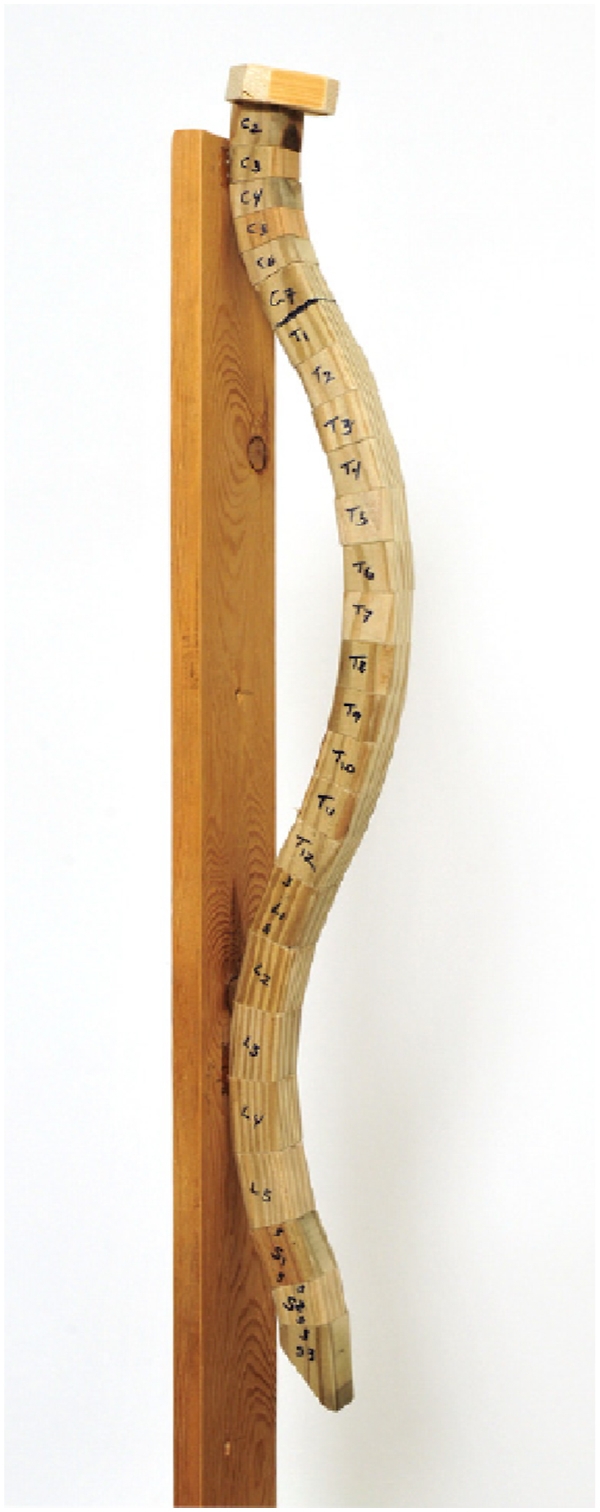
The wooden spine model used for evaluating intra and inter-examiner reliability [55].

Regardless of the effect of high-heels on women's natural difference from the theoretically optimal angle of lumbar curvature, wearing high-heels will increase the models' attractiveness by increasing lumbar lordosis, but there is always concern about the effects of wearing these shoes on the dynamics of the human body and increasing the risk of back pain and lower limb diseases in the users of these shoes; therefore, this paradox between beauty and health is always one concern of young women [54]. In a study [55], lumbar lordosis measurements were carried out using a spinal mouse device. Initially, measurements were captured while the test subjects were barefoot, followed by another set of measurements with the subjects wearing 3 or 4-inch high-heeled shoes after engaging in walking, sitting, and standing activities while wearing the shoes (Fig. 17). The reliability of the spinal mouse measurements was validated using a wooden model (designed to simulate the human spine's proportions) (Fig. 18). The outcomes obtained from assessing body posture using the SonoSense Monitor Analyzer during slow walking at various speeds revealed that wearing high-heels leads to a reduction in lumbar and cervical spine lordosis while simultaneously increasing thoracic spine kyphosis [56]. Early research has established significant increase in muscle activation, particularly in the rectus femoris, soleus, and peroneus longus muscles, when altering the shoe's height [57]. More recent studies confirm that high-heeled shoes, in comparison to sports shoes, impose increased loads on the heel, ankle, lower back, and upper back during walking [58]. However, the experience level of individuals in wearing high-heels significantly affects their muscle activation patterns and overall biomechanics. Experienced wearers tend to rely more on their back muscles to maintain spinal posture, while novices depend more on their abdominal muscles [59]. Additionally, wearing high-heels leads to notably higher strain and activation in leg muscles during the stance phase of gait cycle compared to barefoot, potentially prolonged wearing resulting in muscle fatigue [60]. These findings highlight the compounded biomechanical challenges and increased musculoskeletal strain associated with prolonged high-heel use, underscoring the importance of proper footwear in mitigating long-term health risks.

Although high-heels have negative effects on body kinematics and kinetics, there are ways to mitigate these impacts. Studies have shown that utilizing shock absorption insoles in high-heeled shoes significantly enhances women's comfort by reducing pressure on the heel and front foot, as well as diminishing impact force. These insoles also contribute to improved stability of the ankle joint by modifying the activation of muscles such as the TA and GA muscles [61]. Concerning shoe height, while therapeutic orthoses like typical heel-lift orthotics (THOs) have proven effective for triceps surae muscle diseases, ongoing research aims to develop more efficient orthotic replacements [62]. Additionally, recent research has indicated that the use of a backpack can counteract the influence of high-heels on lumbar curvature by potentially shifting the center of gravity backward [59]. However, in overweight individuals or with excessive backpack weight, this method can increase the load on the L4-L5 and L5-S1 spinal discs, leading to higher disc pressure and potential back pain. Therefore, while this approach can be beneficial for some, careful regulation of the backpack's weight is crucial to avoid exacerbating spinal issues. Extended periods of wearing high-heels are associated with heightened peak dorsiflexion and adduction of the forefoot, a decrease in hallux plantar flexion, and a potential risk for developing hallux valgus [63].

#### 4.1.3 Specialized pregnancy footwear

**Fig. 19:**
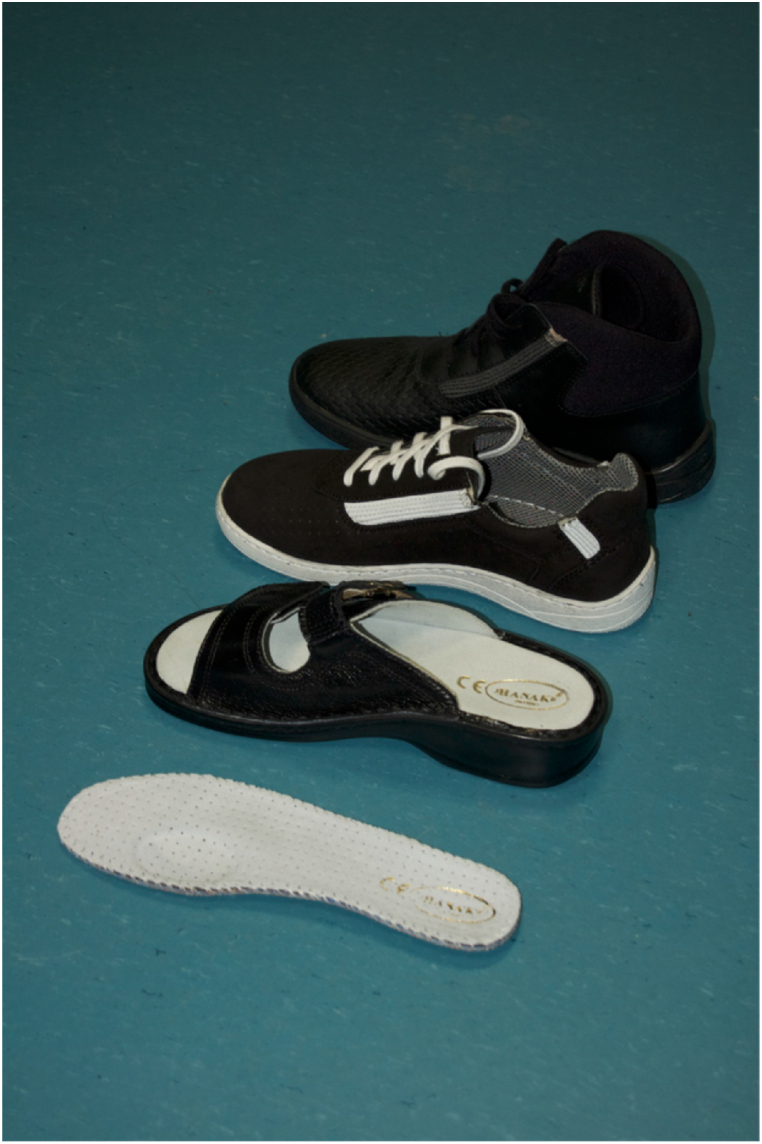
Special footwear and insole used in Gimunová et al.'s study investigating the effects on women during pregnancy and postpartum [64,67].

It appears that due to the alterations in women's body kinematics during pregnancy and the postpartum phase, the development of specialized footwear can significantly aid in achieving optimal kinematics during this period. Gimunova introduced a footwear design tailored for this purpose (Fig. 19) analyzing its effects on women's kinematics during both pregnancy and the postpartum period [64]. These shoes try to help to correct the position of the calcaneus by their three features [65,66]:

1 The shoes feature patented J Hanák R, Ltd. insoles, crafted from pressed cork. Their notable attribute is a depression beneath the first metatarsophalangeal joint, which facilitates more evenly distributed loading of all toes.
2 Elastic leather straps integrated into the shoe's upper sole at the instep and heel sections. These straps provide room for the longitudinal arch of the foot to function effectively.
3 A depression beneath the heel portion of the shoe, aiding in the correction of the calcaneus position.

The utilization of these shoes resulted in considerable straightening of spinal curvature and increased maximum flexion in the hip joint during the postpartum, as opposed to the conditions during pregnancy [67]. Therefore, pregnant women are strongly encouraged to consider such specialized footwear to enhance their comfort, support their changing bodies, and promote optimal musculoskeletal health during and after pregnancy. Adopting these shoes may help mitigate discomfort and improve overall well-being during this transformative time.

#### 4.1.4 Barefoot-inspired shoes

Research indicates that soles tailored to both foot shape and the patient's plantar pressure profile are notably more effective in reducing pressure on the feet compared to soles designed solely based on foot shape [76]. Understanding the distribution of foot pressure during various activities stands as a fundamental aspect of well-designed footwear. By redistributing plantar pressure, footwear serves as a valuable tool in decreasing the risk of foot injuries. Multiple studies have assessed pressure distribution in different shoe types across various activities, as outlined in Table 3. Analyzing these pressure distributions can greatly aid researchers within the footwear field. Yawar et al. [68] suggest that minimal footwear may be more beneficial than highly structured shoes, as it promotes a gait closer to the natural barefoot walking pattern. Overly complex soles can potentially create issues that need to be addressed depending on the individual's injury history. In general, pressure distributions in shoes that mimic the natural gait are likely to be more helpful. However, this guideline may not apply in cases of rehabilitation or specific medical conditions, where more supportive footwear might be necessary [68]. Sinclair et al. [69] conducted a study comparing 3D joint angular kinematics during running in: barefoot condition, conventional cushioned shoes, and barefoot-inspired shoes (footwear designs that aim to mimic the barefoot gait). They discovered that peak flexion was notably higher in conventional shoes compared to running barefoot. However, there was no notable difference in knee joint kinematics between the different footwear conditions. Barefoot running exhibited increased external rotation compared to running in barefoot-inspired shoes, while there was greater ankle plantar flexion in barefoot running compared to shod conditions. Their research highlighted significant differences between barefoot running and running in barefoot-inspired footwear, suggesting that the mechanics of the latter do not accurately replicate those of true barefoot running. However, important parameters such as the runners' experience and the stiffness of the shoes were not considered, which may limit the validity of the study's findings. This underscores the need for further research to develop footwear that more effectively mimics the natural biomechanics of barefoot running.

**Table 3:** Studies about the effect of the shoe on pressure distribution.

Authors	Year of publish	Subjects	Shoe's type	Activity	Result	Methodology
Schaff and Cavanagh [100]	1990	8 males	1- Simple extra-depth shoe 2- Shoe with a 24° rocker bottom design.	Gait (walking)	Rocker-bottom shoes demonstrated a 30 % decrease in peak pressures within the medial and central forefoot and toe regions while showing an increase in pressures in the heel, midfoot, and lateral forefoot areas compared to simple shoes.	A pressure-measuring insole with 72 active elements was used in the right shoe for detailed calibration and measurements.
Jordan and Bartlett [83]	1995	15 males	3 casual shoes	Gait (walking)	Higher total plantar force and force-time integral were associated with decreased perceived comfort. Additionally, decreased comfort on the upper part of the shoe was connected to lower dorsal forces and pressures. However, there was no significant correlation between comfort and overall peak plantar pressure, pressure-time integral, or total plantar area.	A survey was utilized to assess comfort perception. Furthermore, upper foot pressure distribution was measured using a rectangular sensor pad set at 30 Hz, whereas the Mikro-EMED insole, operating at 100 Hz, was employed to measure sole pressure distribution.
Eils et al. [101]	2004	21 males	Soccer shoe	4 soccer-specific movements (normal run (gait), cutting maneuver, sprint, and goal shot)	Increased pressure values were noted during cutting (medial part of the foot), sprinting (first and second ray), and kicking (lateral part of the foot) in contrast to a normal run. However, no notable overall impact of varying surfaces on pressure parameters was observed.	The collection of plantar pressure distribution during movements was conducted using the Pedar Mobile system. This system comprised flexible insoles embedded with 99 sensors, offering a sampling frequency of 50 Hz.
Stewart et al. [102]	2007	6 females and 4 males	1- Flat training shoes 2- MBT shoe	Gait (walking)	MBT shoes demonstrated a 21 % pressure reduction under the midfoot and an 11 % decrease under the heel. However, they showed a 76 % increase in pressure under the toes, resulting in a shift of pressure towards the front of the foot.	A Pedar Ltd. in-shoe system from the UK was utilized to measure both mean and peak pressures in foot areas, in addition to determining the total sole contact area.
Owings et al. [103]	2008	11 females and 11 males	Barefoot and with 3 distinct insoles in a flexible and rocker-bottom shoe.	Gait (walking)	The shape and pressure-based insole used in the flexible shoe showed superior performance in 64 out of 70 regions compared to shape-based insoles. It decreased both peak pressure and force-time integral; however, it led to an increase in force-time integral specifically at the midfoot.	Plantar pressure was measured during barefoot walking using an emed-D pressure platform and by acquiring foam impressions of the feet. Furthermore, in-shoe plantar pressures were recorded using pressure-sensitive arrays from the Pedar-X system under different footwear conditions.
Hagen and Hennig [82]	2009	20 males	NIKE Air Pegasus shoes with different numbers of eyelets (ranging from 1 to 7 pairs) and the tightness of the laces (weak, regular, or tight).	Gait (Running)	Various lacing conditions showed different effects: the seven-eyelet pattern lowered peak pressures under the heel and lateral midfoot, and regular six-eyelet cross-lacing increased loading rates and peak pressures under the heel. perceived comfort remained consistent across lacing patterns, and low lace shoe conditions resulted in reduced impacts and peak pressures under metatarsal heads III and V.	The ground reaction forces, in-shoe pressure distribution, tibial acceleration, and rearfoot motion during running were measured using a piezoelectric force platform from Kistler, piezoceramic transducers from Halm, an Entran accelerometer, and a goniometer with a potentiometer from Megatron.
Hagen et al. [81]	2010	14 males	NIKE Air Pegasus with four lacing patterns (one regular, one tight, and two seven-eyelet lacings (A57, ALL)).	Gait (running)	Subjects perceived the tightest six-eyelet lacing (TIGHT6) and the seven-eyelet lacings (A57, ALL) as the most stable, with A57 being the most comfortable. Peak dorsal pressures were highest with TIGHT6, but the seventh eyelet in A57 and ALL notably reduced pressures on the tarsal bones, aligning with perceived stability and comfort.	Dorsal foot pressures were recorded using a Pedar Insole from Novel GMBH (Munich, Germany). Sensor and eyelet locations were identified through qualitative methods and MRI scans. Evaluation of dorsal pressures in eight anatomical regions was performed utilizing 54 activated sensors embedded in the Pedar sole.
Fiedler et al. [104]	2011	16 females and 4 males	3 shoes with different lacing tightness: 1- Comfortable 2- Loosened 3- Completely loose	Gait (walking)	Alterations in lace tightness resulted in notable pressure adjustments in the hallux, toes 2–5, and the lateral midfoot; however, there was no considerable impact observed on peak or average pressure. Participants noted heightened heel slippage and increased foot mobility when the laces were loosened.	Plantar pressures were measured utilizing the Pedar1-X in-shoe system, while perceived in-shoe displacement was evaluated utilizing a numerical rating scale.
Hillstrom et al. [29]	2013	25 early walkers	1- Barefoot 2- UltraFlex 3- MedFlex 4- LowFlex 5- Stiff	Gait (walking)	The Stiff shoe demonstrated the least peak pressures, whereas the UltraFlex shoe exhibited the highest peak plantar pressures. Additionally, in numerous regions of the plantar foot, the pressures from the UltraFlex shoe closely resembled those experienced during barefoot loading, making them indistinguishable in most areas.	The emed-x system employed capacitance-based force transducers and a 12-region anatomical masking algorithm to measure barefoot plantar pressures. Conversely, the Pedar-X system gathered in-shoe plantar pressures using capacitance-based transducers across various shoes.
Stewart et al. [105]	2014	3 females and 33 males	1- Footwear owned by participants. 2- research footwear chosen by participants, including: a) 21 shoes with good characteristics. b) 15 shoes with poor characteristics.	Gait (walking)	When compared to the shoes owned by participants, shoes with favorable attributes demonstrated a decrease in peak pressure at metatarsals 3 and 5, but concurrently led to heightened pressure time integrals in the midfoot. In contrast, shoes with unfavorable characteristics amplified peak pressure beneath the heel and lesser toes, while diminishing pressure time integrals in the midfoot.	Plantar pressure readings were obtained using the F-Scan Mobile system, which comprises insoles containing 960 pressure-sensing locations. Data collection involved a five-stride protocol, and analysis was performed using the F-Scan software. Spatial and temporal parameters of gait were measured utilizing the GAITMAT II TM, which incorporates six sensor arrays.
Lam et al. [106]	2017	20 males	Footwear featuring midsoles with varying hardness levels (60 and 50 Shore C).	Running (gait), maximal forward sprinting, maximal 45° cutting and lay-up	Lower peak pressure and pressure-time integral across multiple plantar regions during various activities were observed in soft shoes when compared to hard shoes.	The measurement of plantar pressure distribution was conducted using the Pedar Mobile System, which features 99 sensors. Additionally, the capture of shoe-ground angles at touchdown was accomplished through a high-speed camera, while speed measurement during the running task was facilitated by infrared timing gates.

#### 4.1.5 Anti-pronation footwear

**Fig. 20:**
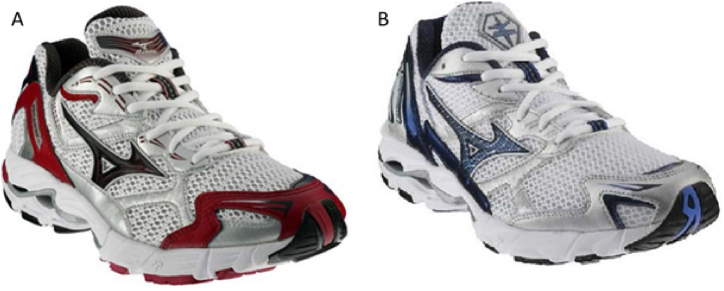
Rose et al.’s shoes: (A) Motion control shoe: Mizuno Wave Inspire (anti-pronation). (B) Neutral shoe: Mizuno Wave Rider [71].

Pronated feet is a condition where the feet roll excessively inward and downward when walking or running. This condition can lead to pain and discomfort in various parts of the body, including the feet, ankles, knees, hips, and lower back. Injuries such as plantar fasciitis, shin splints, and stress fractures can also result from overpronation. Pronation in feet often results from a blend of influences. Factors such as genetic predisposition, insufficient strength in the foot muscles, and wearing inappropriate footwear may collectively contribute to the occurrence of overpronation [11]. To reduce the risk of protonation, anti-pronation footwear is an effective solution. These shoes feature a reinforced heel counter and denser midsole, which help control excessive foot protonation (Fig. 20). Studies showed that anti-pronation shoes (Fig. 20-A) diminish tibial internal rotation during running [70,71] and demonstrate notably higher peak ankle inversion angles and reduced peak eversion angles compared to neutral shoes (Fig. 20-B). Furthermore, these shoes contribute to lower peak moments and powers in lower limb joints, enabling runners to enhance rear foot eversion control. Research has indicated that footwear designed to counter protonation tendencies can diminish peak ankle dorsiflexor and knee extensor moments during running when contrasted with neutral shoes. Consequently, utilizing such footwear may potentially alleviate strain on the ankle and knee joints during physical activities, making them advantageous for individuals engaged in active pursuits [72]. Although these shoes are predominantly designed for women, research suggests they may also offer benefits for men [73]. However, existing studies have certain limitations. For example, they do not isolate factors such as shoe size or specific mechanical properties, and they often exclude professional long-distance runners, who may experience muscle fatigue. These shoes could potentially be beneficial for this group as well.

#### 4.1.6 Gender-specific shoes

Differences in injury risks between men and women during long-distance running highlight the necessity for gender-specific shoe development. Biomechanical studies reveal distinct differences in running patterns between genders. Females often exhibit higher levels of knee abduction, knee internal rotation, and ankle eversion, while males typically demonstrate greater hip flexion. Notably, a significant gender-based variation occurs in the coronal plane during toe-off, with males showing abduction and females showing adduction. These findings suggest that females might benefit from choosing running shoes designed to address coronal plane ankle eversion [74].

#### 4.1.7 Running shoes

Achieving optimal kinematics with minimal energy consumption and maximum efficiency is a primary goal for speed and endurance athletes, highlighting the significance of footwear selection. In races, athletes commonly utilize racing-flat and cushioned running shoes. A comparative analysis between these two types of shoes showed differences in ankle movement patterns. Cushioned shoes demonstrated a notable increase in the ankle dorsiflexion angle upon initial foot contact and maintained a consistently higher maximal ankle dorsiflexion angle throughout the whole running stride. Furthermore, in contrast to racing-flat shoes, cushioned shoes provided a lower ankle plantarflexion angle at toe-off. The consideration of joint work is vital yet frequently neglected when assessing human dynamics in various footwear. Regardless of the specific characteristics or differences between the racing-flat and cushioned shoes, there was a common alteration in the distribution of the positive joint work among the lower extremity joints during running. Moreover, it was noted that racing-flat shoes led to increased negative ankle joint work compared to cushioned shoes [75].

Running shoes are advertised with varying midsole thicknesses, aiming to prevent injuries; however, their effectiveness depends on individual considerations. Early studies exploring the impact of midsole thickness on running biomechanics presented conflicting findings, likely due to the limited scope of thickness examined and the use of diverse shoe models with differing designs and materials [76,77]. In a 2018 investigation by Law et al. [78], the influence of midsole thickness in running shoes on distance runners was analyzed. The study revealed that thinner midsoles led to increased vertical loading rates and reduced contact time compared to thicker midsoles. Notably, no significant differences were observed in footstrike angle, cadence, or stride length among the shoe conditions. The aim of incorporating stiff elements into footwear was to minimize energy loss at the metatarsophalangeal joint, thereby enhancing running performance. This was achieved by integrating flat, stiff carbon-fiber plates along the length of the midsole to restrict dorsiflexion and plantarflexion at the joint while maintaining a low weight [18]. However, optimizing the thickness of these elements is essential before use. Song et al. [79] developed a FE model to simulate complex interactions between the foot and sport shoes and validated it using participants' medical CT images and 3D gait analysis. They later used this model to explore how carbon fiber plates embedded in running shoes influence performance, focusing on different thicknesses (1 mm, 2 mm, and 3 mm) and placement locations (i.e., beneath the insole, within the midsole, and above the outsole). The results showed that thicker plates consistently lowered peak plantar pressure and compressive strain in the forefoot, with the most significant pressure relief occurring when the plate was placed just above the outsole [80]. These studies suggest that incorporating thicker midsoles in running shoes may effectively reduce pressure without increasing metatarsal stress. However, a limitation is that they do not isolate the effect of stiffness. To better understand the specific role of midsole thickness, future studies could employ different midsole structures that maintain consistent stiffness across varying thicknesses, providing more accurate insights into the impact of thickness alone.

**Fig. 21:**
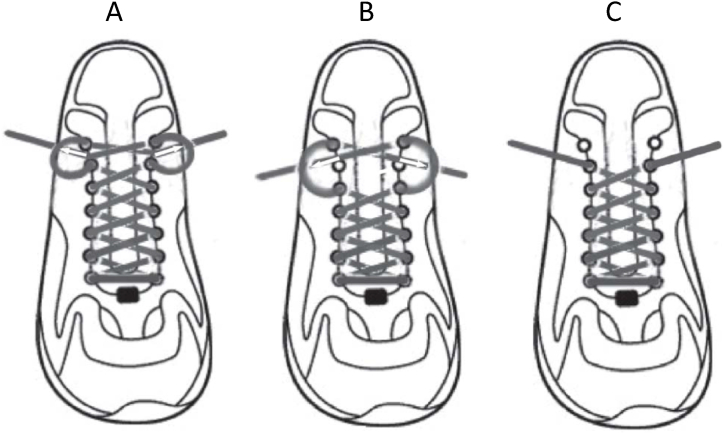
Hagen et al. lacing conditions: (A) seven eyelets ALL, (B) seven eyelets A57 and (C) six eyelets TIGHT6 and REQ6 [81].

The shoelace is an important component of the running shoe's dorsum, and its identification is necessary as it can significantly impact various aspects of the shoe's functionality. Therefore, if researchers fail to isolate this parameter in their research, they cannot observe the effects of the main biomechanical and mechanical parameters. Hagen et al. [81] found that both seven-eyelet lacing systems, specifically All (Fig. 21-A) and A57 (Fig. 21-B), notably enhanced perceived stability and foot-to-shoe connection compared to a standard six-eyelet technique (Fig. 21-C). The study revealed that comfort is closely associated with peak dorsal pressures above the area closest to the lacing, indicating that certain seven-eyelet lacing patterns, like A57, could reduce these pressures while maintaining stability. Hence, it is recommended that running shoes generally incorporate seven eyelets to offer runners a broader range of lacing patterns, accommodating individual preferences. Additionally, understanding the location of peak dorsal pressures may be crucial for developing innovative tongue designs and lacing technologies aimed at improving comfort in running shoes. Furthermore, Hagen and Hennig [82] suggested that runners benefit from tightly or forcefully laced shoes to limit protonation velocity and shock. Participants in the study rated the highest lacing condition as equally pleasant as the standard X-lacing method. Moderate tightness in higher lacing was identified as a comfortable state, effectively connecting the foot and shoe. Conversely, shoes with very low heels and excessively tight laces were disliked by subjects. As per participant feedback, a comfortable shoe is characterized by moderate tightness. Pressure distribution measurements on the foot's dorsum highlighted the variations among different lacing conditions [81,83]. This insight can inform the development of lacing techniques that balance comfort and functionality in footwear design.

### 4.2 Squat

#### 4.2.1 Weightlifting shoes

**Fig. 22:**
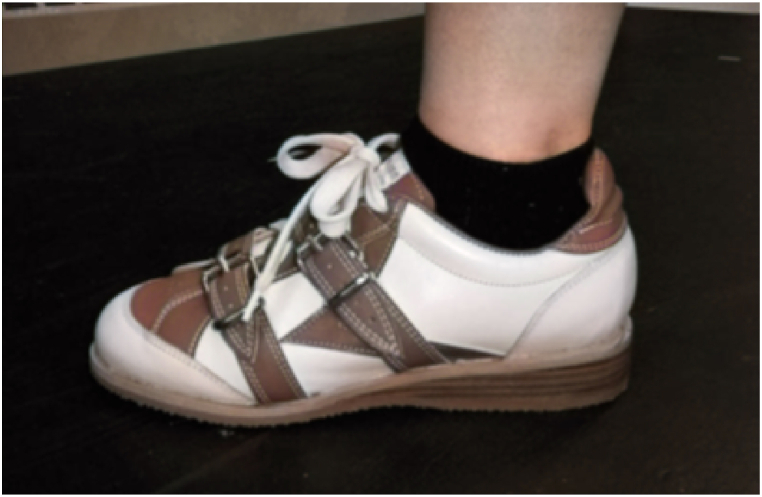
Sato et al. weightlifting shoes [84].

In sports such as weightlifting, the posture of the human body is in a critical state, which increases the risk of injury in these sports. According to articles by Sato et al. [84,85], utilizing weightlifting shoes during the barbell back squat (Fig. 22) may not change the peak thigh flexion angle compared to conventional shoes. However, they found that weightlifting shoes resulted in less trunk lean and greater foot angle, making them essential for weightlifters who want to optimize their performance and reduce the risk of injury. In the study conducted by Whitting et al. [86], investigating the impact of running shoes (RS) versus specialized weightlifting shoes (WS) on barbell back squats at varying percentages of one repetition maximum, the RS condition showed notably higher dorsiflexion compared to the WS condition. However, the main effects analysis revealed no significance for various kinematic parameters such as minimum knee flexion angle, thigh inclination angle, hip flexion angle, and peak trunk lean angle. In Lee et al.'s research [87], subjects engaged in barbell back squats while barefoot on a flat surface, barefoot on a heel-raised platform, and wearing weightlifting shoes with an elevated heel. Although no significant alternation was observed in thoracic, lumbar, and knee kinematics, both heel-raised postures showed a notably higher peak knee flexion compared to the flat condition. These studies highlight that weightlifting shoes can play a pivotal role in optimizing performance and minimizing injury risk during weightlifting, especially by influencing specific kinematic factors.

Besides, experience level can influence muscle activity and resulting kinematics; that is, experienced wearers may require different shoe characteristics than novices to optimize performance and reduce the risk of injuries during squats. In Hayley et al.'s study [88], 32 individuals of different skill levels underwent examination while executing barbell back squats wearing various types of footwear. The research revealed that using weightlifting shoes resulted in a more vertical trunk position, increased knee flexion, and reduced ankle flexion compared to athletic shoes, particularly under an unloaded condition. Experienced individuals achieved deeper squats than novices. When under load, novices exhibited notable disparities in trunk angle and anterior knee positioning with different shoes. Weightlifting shoes were found to enhance squat technique in experienced individuals, although novices might need time to adapt to their use. However, the results of this study contrast with those of Sayers et al. study [89], which found no significant differences in hip range of motion (ROM), knee ROM, thoracic curvature, or lumbar curvature across different footwear types and load conditions. These discrepancies may be due to the lack of control over parameters such as sole stiffness, sole hardness, or outsole structure. Sayers et al. [89] also revealed that novice weightlifters exhibited greater moments around the L4/L5 disk than regular weight trainers during the last 20 % of the squat cycle under level floor conditions. Interestingly, this difference became insignificant during the elevated heel condition. Hence, from a spinal safety standpoint, beginner weight trainers might find some advantage in slightly elevated heels during back squats, whereas regular trainers seem to gain limited benefits from elevated heel shoes during such exercises.

The reviewed articles in this subsection highlight the complexity of understanding weightlifters' kinematics during squats, with differing opinions largely stemming from variations in parameters such as sole hardness and the structural design of various shoe components [38]. Despite these discrepancies, there is a general consensus that beginners should gradually adapt to weightlifting shoes to prevent overcompensation and foster proper technique development. For experienced lifters, these shoes provide substantial advantages by optimizing squat mechanics, enhancing performance, and reducing the risk of injury. Ultimately, the choice of footwear should be tailored to each individual's experience level and biomechanical needs, particularly during high-demand movements like squats.

### 4.3 Drop vertical jump

It seems that one of the reasons for back pain is engaging in highly demanding activities like landing and jumping. Excelling in these movements, crucial in various sports such as basketball, necessitates the use of appropriate footwear [90]. During landing, athletes endure vertical reaction forces several times their body weight [91]. A significant role of footwear is to minimize impact on the ground [92]. Proper shoe design stands as a potential method to help safeguard players prone to injuries stemming from jumping [13,14,93].

#### 4.3.1 Auxetic shoes

**Fig. 23:**
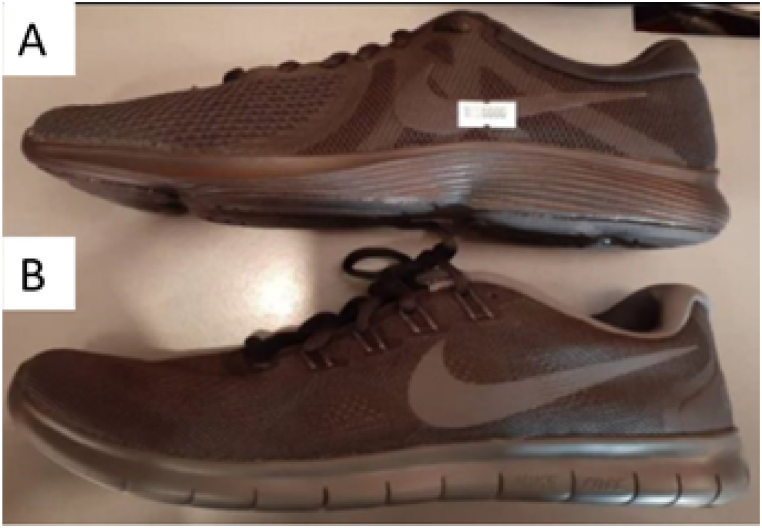
Rahmani et al. footwear: (A) Conventional running shoe, (B) auxetic Nike Free RN shoe [97,98].

The sports industry is using Auxetic materials, which expand laterally under tension and contract under pressure [94,95], in their commercial products to reduce injury risk without compromising athletic performance. Current research suggests that Auxetic materials have the potential for personal protective equipment in sports clothing with advanced features such as better energy absorption and reduced thickness [19]. Nike launched a shoe with an Auxetic sole in 2017, recognizing these advantages [96]. Rahmani et al. [97,98] studied the biomechanics of 11 healthy individuals during a drop vertical jump test comparing regular shoes (Fig. 23-A), Nike Auxetic shoes (Fig. 23-B), and barefoot. The use of auxetic shoes improved maximal hip, knee, and ankle flexion angles. These shoes decreased the maximum shear force and external moment of the lumbosacral disc, minimized the distance from the center of pressure to the heel and reduced the average activity of the longissimus and iliocostalis muscles during the after-landing phase. The shoes examined in this article varied not only in midsole structure but also in sole hardness, sole anatomy, and the composition of different sole layers. Consequently, pinpointing the observed effects solely on the auxetic structure of the shoe sole became challenging [97,98].

#### 4.3.2 Basketball shoes

Alonzo et al. [38] investigated the impact of basketball shoes with 50 and 60 Asker C hardness on the lower extremity biomechanics of 18 individuals during drop vertical jumps. Notably, basketball shoes with softer midsoles showed higher forefoot peak force, along with reduced rearfoot peak force, lower peak flexion moments at the ankle and hip joints during after landing phase, and increased prelanding (100 ms before touchdown) muscle activation in the rectus femoris and tibialis anterior. The study concluded that the variation in midsole hardness resulted in a shift in prelanding muscle activation strategies, subsequently affecting ground reaction forces and joint loadings during landing [38]. The complex effects of midsole hardness were further demonstrated in a FE model, which revealed that higher midsole hardness reduces stress and strain on the plantar fascia and it also increases the loads on the foot [99]. Therefore, selecting the optimal midsole hardness based on individual characteristics can help mitigate the risk of foot injuries. Additionally, these findings highlight the potential inaccuracies in studies that compare shoe structures with varying levels of sole hardness.

## 5 Conclusions and future work

Based on the information provided in the article and Appendix 1 (abstract of effects of different shoes on kinematics and kinetics of human body), several key conclusions can be drawn:

1. Lattice structures in shoe soles: The choice of lattice structure, such as the Grid or Diamond lattice, has distinct effects on shoe stiffness and stress distribution on the foot. The Grid lattice provides stiffness, while the Diamond lattice promotes uniform stress distribution.
2. Composite loofah sponge for vibration-damping: The composite loofah sponge demonstrates effective vibration damping properties, reducing the impact on the knees and ankles during physical activities.
3. Benefits of using the composite loofah sponge: Incorporating the composite loofah sponge into shoe design offers effective vibration damping properties, leading to minimized impact on the knees and ankles.
4. Superior damping properties of silicone material: Silicone material exhibits a significantly greater reduction in hysteresis area compared to TPE, indicating superior damping properties.
5. VDS midsole design for improved stability: The VDS midsole design shows improved stability, minimal distortion, and a high strength-to-weight ratio compared to the UDS midsole.
6. User perception and insole comfort: Users' perception of footwear cushioning correlates with maximal compression of the shoe, while insole comfort is associated with lower impact peak acceleration.
7. Poron insoles for better impact attenuation: Insole comfort plays a crucial role in user satisfaction, and Poron insoles have been found to offer superior impact attenuation compared to Poron/gel conditions. Therefore, incorporating Poron insoles can provide enhanced cushioning and reduce the peak acceleration of impacts, leading to improved comfort and support for individuals.
8. Long-term risks of wearing high-heels: Prolonged use of high-heels increases the risk of back problems by altering natural spine curvatures and increasing compression force on the L5-S1 disk.
9. Benefits of anti-pronation footwear: Anti-pronation footwear reduces stress on the ankle and knee joints during physical activities, making them beneficial for athletes and active individuals.
10. Unstable shoes for exercising muscles: Unstable shoes, with their increased muscle activity amplitudes, particularly in the TA, PL, LG, and lumbar erector spinae muscles, serve as a good choice for exercising devices in the sports industry.
11. Weightlifting shoes for stability and performance: Weightlifting shoes enhance stability and performance but do not significantly reduce the risk of low back injuries during back squats. They may also lead to increased knee joint moments and variability in the center of pressure.
12. Reduce the risk of injury with auxetic shoes: wearing auxetic shoes during high-demanding activities such as vertical jumps can lead to noticeable improvements, including reduced EMG activities in extensor muscles, smaller anterior-posterior distance between the center of pressure and heel, larger maximal hip, knee, and ankle flexion angles, and decreased load on the lumbar spine, suggesting a potential decrease in musculoskeletal injury risk.
13. Improved stability with the seven-eyelet lacing technique: The seven-eyelet lacing technique, such as A57, enhances stability and foot-to-shoe connection compared to a regular six-eyelet technique, reducing peak dorsal pressures and improving comfort in running shoes.

To address current gaps in research, future studies should prioritize mechanical and biomechanical testing on advanced structural designs, such as auxetic and lattice configurations, as well as carbon fiber plates, aiming to simulate barefoot conditions for general users or refined postures tailored for rehabilitation and medical applications. Additionally, researchers should implement systematic testing by using footwear models that vary a single influential parameter—such as sole structure—to conduct preliminary mechanical tests, thereby validating variables before undertaking detailed biomechanical assessments. Furthermore, footwear selection should be aligned with the user's specific activities and desired performance outcomes. For instance, athletes may benefit from unstable footwear depending on targeted muscle groups or desired stability improvements within a particular movement plane. Women who experience overpronation from extended high heel use may consider anti-pronation footwear to mitigate potential long-term impact on foot mechanics. To provide clarity, we have added the footwear biomechanical testing algorithm and the personalized footwear selection algorithm as follow.

### 5.1 Footwear biomechanical testing algorithm

1. Footwear pre-selection: Defines the physical activity and classifies footwear by insole, midsole and outsole structure and material.
2. Mechanical testing: Based on the application, one or more of the following tests must be conducted, and each footwear item receives a score based on these properties.
  2.1 Compression: Assesses energy absorption in compression.
  2.2 Bending: Measures bending flexibility.
  2.3 Torsion: Evaluates torsional flexibility.
  2.4 Impact: Tests shock resistance.
  2.5 Each footwear receives a score based on these properties.
3. In vivo testing (on top-scoring footwear from mechanical tests): based on the application, one or more of the following tests could be conducted.
  3.1 Gait
  3.2 Squat
  3.3 Drop vertical jump
4. Data analysis: Correlates mechanical and in vivo results to see how well each footwear option performs across activities.
5. Conclusion: Recommends footwear with the best balance of mechanical and in vivo performance for specific physical activities.

### 5.2 Personalized footwear selection algorithm

1. Collecting user information via questionnaire:
  1.1 Common activity: Identifies primary physical activities (e.g., walking, jumping, weightlifting).
  1.2 Experience level: Determines experience level (beginner, intermediate, advanced).
  1.3 Gender: Gives gender for biomechanical considerations.
  1.4 Age: Gives age as it affects foot structure, stability, and joint mobility.
  1.5 General physical characteristics: Records body weight, height and foot structure.
2. Collecting medical reports:
  2.1 Muscle strength assessment: Determines if specific muscles are weaker or require strengthening for injury prevention.
  2.2 Kinematic abnormalities: Detects any abnormal movements, such as overpronation.
  2.3 Joint loads and moments: Evaluates joint loading and moments, especially during common activities, to understand stress areas.
3. Determining footwear requirements based on the collected data:
  3.1 For rehabilitation: Recommends footwear designed for injury support, stability, and motion correction.
  3.2 For performance enhancement:
    3.2.1 Injury prevention: Selects shoes with features that minimize impact forces and stabilize joints.
    3.2.2 Energy return and flexibility: Chooses footwear that offers optimal energy return and flexibility to support agility and performance.
    3.2.3 Stability for heavy loads: For weight-bearing activities, prioritizes footwear with solid soles and arch stability.
4 Recommending a suitable footwear:
  4.1 Cross-reference requirements: Compares user requirements with available footwear options to select shoes that balance support, flexibility, cushioning, and stability.
  4.2 Footwear selection based on activity and goal:
    4.2.1 Rehabilitation: Recommends footwear designed for injury support, stability, and motion correction.
    4.2.2 Performance: Recommends footwear focusing on enhancing performance and lowering injury risks through flexibility, energy return, and joint support.

## CRediT authorship contribution statement

**Mohammad Mahdi Mohammadi:** Writing – original draft, Methodology, Investigation, Data curation. **Amir Nourani:** Writing – review & editing, Supervision, Conceptualization.

## Data availability statement

Data sharing is not applicable. No data was used for the research described in the article.

## Declaration of competing interest

The authors declare that they have no known competing financial interests or personal relationships that could have appeared to influence the work reported in this paper.
