# Supplementary material for: Testing the effects of footwear on biomechanics of human body: A review
Source: Heliyon. 2025 Feb 20;11(4):e42870. doi: 10.1016/j.heliyon.2025.e42870 (PMC11904544; doi:10.1016/j.heliyon.2025.e42870)
Supplement: Multimedia component 1 [file mmc1.docx]

APPENDIX 1

**Studies upon the effects of shoes on kinematics and dynamics of human body.**

| High heels shoes | | | | | | |
| --- | --- | --- | --- | --- | --- | --- |
| Authors | Year of publish | Subjects | Shoe’s type | Activity | Result | Methodology |
| Stefanyshyn et al. [1] | 2000 | 13 females | 1= Enzo Angiolini Liberty shoes (flat)  2- Amalfi Gina shoes (3.7 cm)  3- Amalfi Dahlia shoes (5.4 cm)  4- Caressa Haute shoes  (8.5 cm). | Gait (walking) | The study observed that as shoe height rose, there was a notable increase in active vertical, propulsive, and braking forces. Additionally, higher ankle and knee flexion, as well as increased activity in the soleus and rectus femoris muscles, were noted with greater heel heights. Notably, the shoe with a 3.7 cm heel height exhibited the highest vertical impact force peaks and maximal vertical loading rates, whereas the flat shoe and the one with an 8.5 cm heel height showed comparatively lower values. | The study collected kinetic data utilizing a Kistler force platform (Switzerland), while simultaneously capturing kinematic data using a Motion Analysis 4 camera system. EMG activity from gastrocnemius, soleus, peroneus longus, tibialis anterior, rectus femoris, semitendinosus, biceps femoris, and vastus medialis muscles was recorded using a surface electrode EMG system. |
| Russell et al. [2] | 2012 | 50 people (32 females and 18 males in test group and 3 females and 6 males in control group) | Barefoot with 3- or 4-in high-heeled shoes | Standing posture | high-heeled shoes did not affect lumbar lordosis while standing. | A Spinal Mouse was used to measure lumbar lordosis in test participants. |
| Wang et al. [3] | 2018 | 15 females | 1- High heels  2- Barefoot | Gait (walking) | Wearing high heels was associated with an increase in hallux dorsiflexion and a decrease in hallux plantarflexion during the initial stance phase. Additionally, heightened forefoot adduction was noted. Regarding the hind foot, there was an elevation in dorsiflexion in the horizontal plane, greater internal rotation, and a reduction in peak hind foot extension rotation. These findings suggest a potential heightened risk of foot injuries associated with wearing high heels. | A Vicon motion system with 8 cameras (Oxford Metrics Ltd., Oxford, UK) was used to capture kinematic data of the hallux, forefoot, and hind foot. |
| Schroeder and Hollander [4] | 2018 | 37 females | High-heels (7–11 cm) were compared to standard control shoes | Standing posture and Gait (walking) | wearing high heel shoes results in a reduced pelvic tilt and increased transversal pelvic rotation, indicating moderate effects on pelvic alignment. | The study used video rasterstereography to analyze spine shape, providing high-resolution 3D reconstructions. |
| Derlatka and Bogdan [5] | 2018 | 99 people | 1- High heels  2- Sport shoes | Gait (walking) | The study illustrated the capacity of accelerometers and pressure sensors to distinguish between individuals wearing sports shoes or high heels based on their gait patterns. | Using force plates made by the Kistler Company and Microsoft Kinect v2 for gathering data. |
| Jandova et al. [6] | 2019 | 30 females | 1- Flat shoes (FS)  2- High heels | Gait (walking) | The wearing of high heels resulted in changes to plantar pressure and spinal curvature, which persisted even with a decrease in walking speed. | The Pedar-X (Novel, Munich, Germany) measuring insoles were used to measure the plantar pressure distribution. For postural measurement, the sonosens Monitor Analyzer (Gefremed, Chemnitz, Germany) was used. |
| Lee et al [7] | 2019 | 7 females and 7 males | 1- Barefoot on a flat surface  2- Barefoot on a heel-raised platform  3- Heel-raised weightlifting shoes | Barbell back squats | Elevated heel foot positions did not produce significant impacts on spinal and knee extensor muscle activations, nor on trunk and knee kinematics. Consequently, heel-raised weightlifting shoes are unlikely to offer protection against back injuries for recreational weightlifters during the barbell back squat. | Motion data were captured using a 12-camera 3D system (Vicon Bonita cameras; Vicon Motion Systems, Oxford, UK). A wireless spring electrogoniometer (Delsys Trigno Biaxial Goniometer; Delsys Inc., Natick, MA, USA) was placed on the lumbar spine at the L3 level to track segmental kinematics. 3 wireless EMG transceivers (Delsys Trigno Wireless System; Delsys Inc., Natick, MA, USA) recorded neuromuscular activity from the right paraspinal and right knee extensor muscles. |
| Sanchez-Gomez et al.[8] | 2020 | 26 healthy, regular recreational runners of both sexes | 1- Typical heel-lift orthotics (THOs)  2- Propulsion heel-lift orthotic (PHO)  3- Sports shoes only (SO) | Gait (running) | A novel PHO may increase muscle activity of the gastrocnemius lateralis; consequently, its prescription to treat triceps surae muscle injuries is cautioned. | EMG activity of the gastrocnemius medialis and lateralis muscles was recorded over 30 seconds using the NeuroTrac Simplex Plus (Verity Medical) EMG device. |
| Sayers et al. [9] | 2020 | 10 novice and 10 regular weight trainers. 5 males and 5 females in each group | back squat | Standing posture | Novice weight trainers may find elevating heels advantageous for performing back squats, whereas experienced weight trainers seem to derive only limited benefits from using either elevated heels or weightlifting shoes. | a motion capture system from Vicon Motion System (UK), synchronized with force platforms from Kistler Instrument AG (Switzerland) used for data analysis. |
| José Pino-Ortega et al. [10] | 2021 | 7 females | 1- High-heel shoes (HHS) with 80-mm heel  2-Sport shoes (SS) with 10-mm drop. | Gait (walking) | At both types of shoes, the greatest force exerted on the accelerometer was experienced at the heel of the body, whereas the lowest values were recorded at the upper and lower back. Significant discrepancies were noted between shoes at all anatomical locations, with higher loads observed with high-heeled shoes. Furthermore, the type of shoe exerted an effect on the change in the laterality profile. | The WIMU PRO inertial device records forces and accelerations at specific anatomical locations on the lower limbs and trunk. |
| Liau et al. [11] | 2021 | 16 females | High heels | Standing posture | the long-term high heels users exhibited significant hyper-lumbar lordosis. Furthermore, the USERS showed significantly greater recruitment of back muscles, but the NON-USERS tended to recruit significantly more abdominal muscles to control their posture. | Muscle activity was recorded using bipolar surface EMG electrodes (BagnoliTM-8, Delsys, Boston, MA, USA), while body kinematics were captured using the OptiTrack motion capture system (Natural Point, Corvallis, OR, USA). |
| Meskó et al. [12] | 2021 | 52 females | 1- High heels  2- Flat shoes | Gait (walking) | Wearing heels as compared to flats would increase young women’s lumbar curvature was confirmed. | The models’ lumbar curvature was measured with a virtual protractor tool (Screen Protractor, Iconico, Inc., Phoenix, AZ, US) |
| Lawrence et al. [13] | 2022 | 30 males | 1-Barefoot  2-Shoes with height-elevating the insole with a height of 5, 7, or 9 cm. | Gait (walking), one leg stand and tandemstand | However, there was no significant difference in gait parameters among shoes. Nonetheless, there was a significant change in balance when comparing shoes with high soles of different heights. | the Shapiro walk test was utilized, and the parametric form of the One-Way ANOVA test and the non-parametric form of the Kruskal-Wallis test were used for data analysis. |
| Yu-Jin Cha [14] | 2022 | 52 females. | 1-7 cm high-heels with the shock-absorbing insoles.  2- 7 cm high-heels without the shock-absorbing insoles. | Gait (walking) | high-heeled shoes with Wearing shock-absorbing insoles improved postural stability, pressure distribution, and comfort. | The Pedar-X System was utilized for in-shoe pressure measurements, the Noraxon EMG system was employed for surface electromyography, Motion Analysis OptiTrack was used for kinematics, and functional mobility was assessed using the Timed Up and Go test (TUG). |

| Unstable Shoes | | | | | | |
| --- | --- | --- | --- | --- | --- | --- |
| Authors | Year of publish | Subjects | Shoe’s type | Activity | Result | Methodology |
| Nigg et al. [15] | 2006 | 3 females and 5 males | 1- An unstable test shoe (Masai Barefoot Technology, MBT)  2- a stable control shoe. | Standing and gait (walking) | the center of pressure excursion was significantly greater in the unstable compared to the control shoe in standing. unstable shoe increased significantly Electromyographic activity for the tibialis anterior. During gait, plantar-dorsiflexion showed a significantly more dorsiflexed position during the first half of the stance in the unstable shoe compared to the stable control shoe. | A high-speed video camera system (Motion Analysis Corporation, USA) equipped with eight cameras recorded kinematic data at 240 Hz. Kinetic data was concurrently collected using a force platform (Kistler, Switzerland) operating. Myoelectric signals were recorded using surface electrodes from Biovision (Germany)." |
| Stewart et al [16] | 2007 | 6 females and 4 males | 1- Flat-soled training shoes  2- MBT shoe | Gait (walking) | MBT shoes reduced pressure under midfoot and heel by 21% and 11% respectively, but increased pressure under toes by 76%, shifting pressure towards the front of the foot. | An in-shoe system (Pedar Ltd., UK) was used to measure mean and peak pressures. |
| Koyama et al. [17] | 2012 | 14 males | 1- Unstable shoes (US; Shape-ups, SKECHERS, USA)  2- normal walking shoes (WS) | Gait (walking) | Unstable footwear might elevate lower leg muscle activity and energy expenditure without affecting the Rating of Perceived Exertion or the optimal walking speed. | Kinematics were recorded using a video camera (Exlim EX-F1, Casio, Japan), capturing contact time, cadence, and step length. EMG signals from various leg muscles were recorded using bipolar surface electrodes (Ambu, Balerup, Denmark). Heart rate was continuously monitored using a Polar device (CS400, Polar Electro, Finland), while VO2 was recorded with a metabolic cart (AE-300S, Minato Medical Science, Japan). Oxygen cost was calculated, and the optimal walking speed was determined. Rating of Perceived Exertion (RPE) was evaluated using a Borg scale (Borg, 1974) after each walking speed. |
| Debbi et al. [18] | 2012 | 10 males | The instability level control device (AposTherapy System) that has three stages:  Stage 0: stable  Stage 1: low instability  Stage 2: low instability." | Gait (walking) | There was a notable increase in stride-to-stride variability (STSV) observed between stages 0 and 1, as well as between stages 0 and 2, across nearly all parameters. Specifically, a significant elevation between stage 0 and both stages 1 and 2 was noted for knee flexion moment, knee varus moment, knee flexion angle, and hip adduction angle. Moreover, there was a significant increase in knee varus moment within STSV between stages 1 and 2 | Kinematic data were collected with a Vicon motion capture system, and dynamic data with a AMTI platform. |
| Buchecker et al. [19] | 2013 | 27 males | 1- Unstable Masai Barefoot Technology (MBT) shoes  2- standard footwear | Standing | Wearing unstable MBT shoes resulted in increased flexion at the mid-thoracic level by 0.8°. Moreover, it induced greater mean velocities of angular displacement at the thoracolumbar region by 11.2% and at the lumbopelvic region by 10.8%, coupled with heightened activity of the lumbar erector spinae muscles by 18.2%. | Kinematic data was collected using an eight-camera motion analysis system (MX13, Vicon, Oxford, UK). Electromyographic (EMG) data was captured using surface electrodes. |
| Souza et al [20] | 2014 | 30 females | 1- Unstable MBT shoes  2- Barefoot. | Long term wearing | Prolonged use of unstable shoes resulted in increased activity of the biceps femoris (BF) muscles, decreased activity of the gluteus maximus (GM) muscles, and an increase in antagonist co-activation accompanied by decreased reciprocal activation. Additionally, there was a reduction in center of pressure (CoP) displacement. | Electromyographic (EMG) data was collected utilizing the Biopac MP 150 Workstation along with silver-silver chloride surface electrodes, while center of pressure (CoP) displacement values were acquired using a Bertec force plate. |
| Gu, Y et al. [21] | 2014 | 22 males | 1- General shoes with flat-sole  2- experimental shoes with some unstable elements are stuck to the outsole of general shoes in the heel and forefoot zone | Gait (Walking) | The experimental shoes were associated with decreases in knee abduction and internal rotation, as well as hip internal rotation. Furthermore, there were reductions observed in ankle inversion and internal rotation amplitudes. | Kinematic analysis was conducted with an 8-camera Vicon motion analysis system from Oxford Metrics Ltd., UK. Simultaneously, surface electromyography (SEMG) measurements were recorded using an eight-channel EMG device from Mega Electronics, Finland. |
| Sousa et al [22] | 2016 | 30 females | 1- MBT shoes  2- Conventional shoes | Standing posture | Wearing unstable shoes resulted in increased CoP displacement variables and decreased co-activation command, suggesting compromised stability and altered muscle activation patterns. | Leg muscle activity was monitored utilizing the Biopac MP 150 Workstation, while stabilometry parameters were measured through a Bertec force plate. |
| Salvador-Coloma et al [23] | 2018 | 18 females and 17 males | 1- Unstable shoe (MBT, model AFIYA 5)  2- Flat sole shoe (John Smith Classic) | Gait (walking) | Wearing unstable shoes notably elevated EMG activity in the erector spinae (ES), rectus abdominis (RA), and oblique internus (OI) muscles, with a particularly pronounced effect size observed for the ES muscle. | Electromyography (EMG) signals from the erector spinae (ES), rectus abdominis (RA), oblique internus (OI), and oblique externus (OE) muscles were recorded using the ME6000s electromyograph. Ankle range of motion data was collected using a twin-axis electronic goniometer. |
| Khoury-mireb et al. [24] | 2019 | 15 males | The instability level control device (AposTherapy System) that has two levels:  1- instability elements attached  2- without instability elements | Gait (Walking) | The results indicate that the unstable device employed in the study could potentially decrease gait variability at the foot, ankle, and shoulders, while simultaneously increasing variability in spine rotation angle. | To capture kinematic data, an 8-camera Vicon motion analysis system (Oxford Metrics Ltd., Oxford, UK) was used to perform 3D motion analysis. GRFs were collected by two AMTI OR6–7-1000 force plates. |
| Zhoe et al. [25] | 2021 | 15 males | 1- normal control shoes  2- bionic unstable footwear | Gait (Running and walking) | The study found significant differences in hip extension and flexion between control and bionic shoes during the walking stance phase. No significant differences were observed in ankle and moment during running. Muscle activity in the rectus femoris, tibialis anterior, and medial gastrocnemius varied during walking, while running showed significant differences in the rectus femoris, tibialis anterior, medial, and lateral gastrocnemius between the two shoe types. | Kinematic data were collected with a Vicon motion capture system, and dynamic data Hz with a Kistler force platform. A Delsys EMG system recorded muscle activations, including the gastrocnemius (medial and lateral), vastus medialis, vastus lateralis, rectus femoris, and tibialis anterior. |
| Zhoe et al. [26] | 2021 | 15 males | 1- normal control shoes  2- bionic unstable footwear | single-leg landing | Their findings showed significant differences in knee and hip flexion-extension angles, ankle dorsiflexion-plantarflexion moments, ankle eversion-inversion, and knee abduction-adduction angles across planes. | Kinematic data were collected with a Vicon motion capture system, and dynamic data with a Kistler force platform. |

| Pregnancy | | | | | | |
| --- | --- | --- | --- | --- | --- | --- |
| Authors | Year of publish | Subjects | Shoe’s type | Activity | Result | Methodology |
| Gimunová et al. [27] | 2017 | 30 females | 1- Slippers Sneakers Winter shoes with custom design.  1- Normal Slippers Sneakers Winter shoes. | Gait (walking) | Wearing custom footwear may potentially prevent a decrease in venous blood velocity during the later stages of pregnancy. | During each data collection session, assessments of peak systolic blood flow velocity and the cross-sectional area of the right popliteal vein were undertaken using a MySonoU6 ultrasound system. |
| Gimunová et al. [28] | 2020 | 41 females | 1- Slippers Sneakers Winter shoes with custom design.  1- Normal Slippers Sneakers Winter shoes. | Gait (walking) | Custom footwear appears to play a role in preventing foot arch collapse, as evidenced by the observed changes in the kinematic gait pattern. Conversely, the regular slippers led to alterations indicative of foot arch collapse and hindfoot hyperpronation during late pregnancy and postpartum, including increased knee flexion and spinal curvature. | The gait kinematics were recorded using the Simi Motion System. |

| Weightlifting shoes | | | | | | |
| --- | --- | --- | --- | --- | --- | --- |
| Authors | Year of publish | Subjects | Shoe’s type | Activity | Result | Methodology |
| Fortenbaugh et al.[29] | 2010 | 20 males | 1- Running shoes (RS)  2- Weightlifting shoes (WS) | Barbell back squats | The use of WS shoes appeared to encourage a more upright stance, with less displacement of the bar and hip. This indicates a potential for WS to promote a safer and more efficient squatting technique by facilitating a more erect trunk posture. | A Panasonic digital camera at 60 Hz (Osaka, Japan) was utilized to capture kinematic data. |
| Sato et al. [30] | 2012 | 20 males and 5 females | 1- Running shoes (RS)  2- Weightlifting shoes (WS) | Barbell back squats | Wearing WS shoes reduced overall trunk lean and increased foot segment angle during back squats, possibly contributing to greater muscle excitation in knee extensors. | A Panasonic digital camera at 60 Hz (Osaka, Japan) was utilized to capture kinematic data. |
| Whitting et al. [31] | 2016 | 9 males | 1- Standard sports trainers (running shoes (RS))  2- Weightlifting shoes (WS) | Barbell back squats | The RS condition exhibited significantly more dorsiflexion compared with the WS condition in both ankles. | High-speed cameras from Vicon (Oxford, UK) recorded kinematics, while Kistler force plates (Winterthur, Switzerland) recorded GRF data. |
| Legg et al. [32] | 2017 | 20 males | 1- Weightlifting shoes (WS)  2- athletic shoes (AS) | Barbell back squats | In loaded conditions, WS led to reduced ankle flexion and increased knee flexion compared to AS, along with a more upright trunk posture and greater knee moment in the unloaded condition. Additionally, experienced participants showed a significantly greater increase in knee and hip flexion with WS than novices in the unloaded condition. | 11 cameras from Vicon (Oxford, UK) recorded kinematics. |

| Anti-pronation shoes | | | | | | |
| --- | --- | --- | --- | --- | --- | --- |
| Authors | Year of publish | Subjects | Shoe’s type | Activity | Result | Methodology |
| Clarke et al. [33] | 1983 | 10 subjects | 36 different shoes in combinations of three midsole harnesses, three heel flares, and four heel heights. | Gait (running) | The study found that shoes with soft midsoles allowed significantly more maximum pronation (MP) and total rearfoot movement (TRM) than those with either medium or hard midsoles, while shoes with a 0-degree heel flare enabled more MP and TRM than those with either 15-degree or 30-degree heel flares. Interestingly, heel height did not show a significant effect on either MP or TRM. | high-speed movie film (photosonics cine camera) was taken from the rearfoot. |
| Cheung and Ng [34] | 2007 | 25 females | 1- latest model of motion control shoes (Supernova control, Adidas)  2- neutral shoes (Supernova cushion, Adidas) | Gait (Running) | In neutral shoe testing, a significant 6.5° increase in rearfoot angle occurred with muscle fatigue, while motion control shoe testing showed rearfoot angle insignificance before and after fatigue. Moreover, rearfoot motion during running with neutral shoes surpassed that with motion control shoes in both pre-and post-fatigue states, indicating motion control shoes' efficacy in managing over-pronation regardless of muscle fatigue. | 3 cameras from Vicon (Oxford, UK) recorded kinematics |
| Rose et al. [35] | 2011 | 24 subjects. | 1- neutral shoes  2- motion control shoes | Gait (running) | A disparity in tibial rotation in the transverse plane was observed between a motion control running shoe and a neutral running shoe. | the Codamotion 3-D movement analysis system measured the total range of proximal tibial rotation. |
| Alizadeh et al. [36] | 2024 | 26 subjects | 1- with anti-pronation insole  2- without anti-pronation insole | Gait (running) | The study found significant changes in the activity of the quadriceps, hamstrings, gluteus medius, and gastrocnemius muscles with the use of anti-pronation insoles in individuals with anterior cruciate ligament (ACL) reconstruction and pronated feet, indicating potential clinical benefits. | EMG system (Data LITE EMG, Biometrics Ltd., England) was used to measure the activity of the tibialis anterior, gastrocnemius medialis, vastus medialis, vastus lateralis, rectus femoris, biceps femoris, semitendinosus, and gluteus medius muscles. |

| Running Shoes | | | | | | |
| --- | --- | --- | --- | --- | --- | --- |
| Authors | Year of publish | Subjects | Shoe’s type | Activity | Result | Methodology |
| Sinclair et al. [37] | 2012 | 12 females and 12 males. | Saucony Pro Grid Guide 2 | Gait (running) | Females exhibited significantly greater knee abduction, knee internal rotation, and ankle eversion, whereas males displayed notably higher hip flexion. | Kinematic data was recorded at using an eight-camera motion analysis system from Qualisys Medical AB (Goteburg, Sweden), while axial accelerations at the tibia were measured using a tri-axial accelerometer from Biometrics ACL 300 (UK), securely attached to the tibia. |
| Chambon et al. [38] | 2014 | 15 males | barefoot and with five shoes of different midsole thickness (0 mm, 2 mm, 4 mm, 8 mm, 16 mm) | Gait (running) | While no significant effect of midsole thickness was observed on GRF and tibial acceleration, an increase in contact time was noted with thicker midsoles. Additionally, barefoot running induced ankle plantar flexion at touch-down, higher ankle dorsiflexion, and lower knee flexion during the stance phase compared to shod running. | kinematics was recorded using an optoelectronic motion capture system equipped with eight cameras, while GRF was measured by a force platform (Kistler 9281 CA), and tibial acceleration was monitored using a tri-dimensional accelerometer fixed on the subjects' skin. Data were collected. |
| Squadrone et al. [39] | 2015 | 14 males | 1- barefoot  2- cushioned stability shoe (Saucony progrid Guide)  And five different shoes marketed as minimalist by their producers, including 3- Newton Running MV2  4- New Balance MR00GB  5- Nike Free 3.0V4  6- Inov8 Bare-X 200  7- Vibram fivefingers Seeya  8- Saucony Kinvara2. | Gait (running) | The research revealed notable differences across various parameters of footwear models, encompassing mass, stack height, heel-to-toe drop, shock absorption, and flexibility. These variances translated into changes of 1.7% to 7.5% in stride length, step time, and contact time when contrasting barefoot running with shod conditions. Moreover, barefoot running elicited a stride frequency increase of 1.6% to 4.1%. The findings suggest that minimalist footwear, characterized by lower heel heights and reduced shock absorption, more closely approximates the biomechanics of barefoot running. | The Zebris FDM-T (Germany) instrumented treadmill was used to measure the pressure distribution at the foot–ground or shoe–ground interface. |
| Law et al. [40] | 2019 | 15 males | Shoes with midsole thickness, from 1 mm to 29 mm | Gait (running) | Thinner midsole shoes (1- and 5-mm) were found to notably elevate vertical loading rates and decrease contact time compared to thicker midsole counterparts (25- and 29-mm). | an 8-camera Vicon motion system (Oxford, UK) operating was employed, synchronized with kinetic data sampled. |
| Sanno et al. [41] | 2021 | 18 males | 1- racing flat shoes  2= cushioned running shoes | Gait (running) | Regardless of shoe design, alterations in running mechanics occurred within the first 2 km of the run, suggesting habituation rather than fatigue effects. However, researchers did not find a difference between shoes in the fatigue-related redistribution of joint work from distal to proximal joints. | A 13-camera Vicon motion system (Oxford, UK) was employed, synchronized with GRF data collected from four force transducers embedded in a single-belt treadmill (Treadmetrix, Park City, USA). |

| Auxetic | | | | | | |
| --- | --- | --- | --- | --- | --- | --- |
| Authors | Year of publish | Subjects | Shoe’s type | Activity | Result | Methodology |
| Dehaghani et al. [42] | 2022 | 11 males | Barefoot, conventional running shoes, and auxetic Free RN running shoes | Drop vertical jump | Results indicate that using auxetic shoes can reduce the load on the lumbar spine during high-demanding activities such as vertical jumps and thus may decrease the musculoskeletal risk of injuries during these activities. | A 10-camera Vicon motion capture system (Oxford, UK) operating at 120 Hz recorded kinematics, while GRF and center of pressure (COP) data were simultaneously recorded using two adjacent force platforms (Kistler Instrument AG, Switzerland). Additionally, electromyography (EMG) activities of the main trunk extensor and flexor muscles were captured using an eight-channel wireless system (Myon 320, Switzerland) at 1200 Hz. |

| Midsole Hardness | | | | | | |
| --- | --- | --- | --- | --- | --- | --- |
| Authors | Year of publish | Subjects | Shoe’s type | Activity | Result | Methodology |
| Alonzo et al. [43] | 2020 | 18 males | Basketball shoes of different midsole hardness (50, 60 Asker C) | Drop vertical jump | Modifying midsole hardness led to variations in pre-landing muscle activation strategies, consequently affecting the magnitudes of GRF and joint loadings during landing. | An 8-camera motion capture system (Motion Analysis Corporation, Santa Rosa, CA, USA) recorded lower body kinematics, while GRF data were simultaneously recorded using two adjacent force platforms (OR6-7-2000, Advanced Mechanical Technology Inc., Watertown, MA, USA). |

# References

1. Stefanyshyn, D.J.; Nigg, B.M.; Fisher, V.; O’Flynn, B.; Liu, W. The Influence of High Heeled Shoes on Kinematics, Kinetics, and Muscle EMG of Normal Female Gait. *J. Appl. Biomech.* **2000**, *16*, 309–319.

2. Russell, B.S.; Muhlenkamp, K.A.; Hoiriis, K.T.; DeSimone, C.M. Measurement of Lumbar Lordosis in Static Standing Posture with and without High-Heeled Shoes. *J. Chiropr. Med.* **2012**, *11*, 145–153, doi:10.1016/J.JCM.2012.02.002.

3. Wang, M.; Gu, Y.; Baker, J.S. Analysis of Foot Kinematics Wearing High Heels Using the Oxford Foot Model. *Technol. Heal. care* **2018**, *26*, 815–823.

4. Schroeder, J.; Hollander, K. Effects of High-Heeled Footwear on Static and Dynamic Pelvis Position and Lumbar Lordosis in Experienced Younger and Middle-Aged Women. *Gait Posture* **2018**, *59*, 53–57, doi:10.1016/J.GAITPOST.2017.09.034.

5. Derlatka, M.; Bogdan, M. Recognition of a Person Wearing Sport Shoes or High Heels through Gait Using Two Types of Sensors. *Sensors* **2018**, *18*, 1639.

6. Jandova, S.; Gajdoš, M.; Urbanová, K.; Mikuľáková, W. Temporal and Dynamic Changes in Plantar Pressure Distribution, as Well as in Posture during Slow Walking in Flat and High-Heel Shoes. *Acta Bioeng. Biomech.* **2019**, *21*, 131–138.

7. Lee, S.-P.; Gillis, C.B.; Ibarra, J.J.; Oldroyd, D.F.; Zane, R.S. Heel-Raised Foot Posture Does Not Affect Trunk and Lower Extremity Biomechanics During a Barbell Back Squat in Recreational Weight Lifters. *J. Strength Cond. Res.* **2019**, *33*.

8. Sanchez-Gomez, R.; Becerro-de-Bengoa-Vallejo, R.; Romero Morales, C.; Losa-Iglesias, M.E.; Castrillo de la Fuente, A.; López-López, D.; Díez Vega, I.; Calvo-Lobo, C. Muscle Activity of the Triceps Surae with Novel Propulsion Heel-Lift Orthotics in Recreational Runners. *Orthop. J. Sport. Med.* **2020**, *8*, 2325967120956914.

9. Sayers, M.G.L.; Bachem, C.; Schütz, P.; Taylor, W.R.; List, R.; Lorenzetti, S.; Nasab, S.H.H. The Effect of Elevating the Heels on Spinal Kinematics and Kinetics during the Back Squat in Trained and Novice Weight Trainers. *https://doi.org/10.1080/02640414.2020.1738675* **2020**, *38*, 1000–1008, doi:10.1080/02640414.2020.1738675.

10. Pino-Ortega, J.; Oliva-Lozano, J.M.; Gómez-Carmona, C.D.; Rojas-Valverde, D.; Bastida-Castillo, A.; Moreno-Pérez, V.; Nakamura, F.Y. Impact of High-Heeled and Sport Shoes on Multi-Joint External Load Profile during Walking. *J. Back Musculoskelet. Rehabil.* **2021**, *34*, 389–398.

11. Liau, Y.Y.; Kim, S.; Jin, S.; Ryu, K. The Effect of Wearing High-Heels and Carrying a Backpack on Trunk Biomechanics. *Int. J. Ind. Ergon.* **2021**, *86*, 103229, doi:https://doi.org/10.1016/j.ergon.2021.103229.

12. Meskó, N.; Őry, F.; Csányi, E.; Juhász, L.; Szilágyi, G.; Lubics, O.; Putz, Á.; Láng, A. Women Walk in High Heels: Lumbar Curvature, Dynamic Motion Stimuli and Attractiveness. *Int. J. Environ. Res. Public Health* **2021**, *18*, 299.

13. Lawrence, C.; Novalinda, C.; Chiuman, L.; Girsang, E. Changes In Gait, Balance and Comfort That Is Felt in The Use of Shoes with Height-Elevating Insole with A Height Of 5, 7 and 9 Cm. *Int. J. Heal. Pharm.* **2022**, *3*, 191–196.

14. Cha, Y.-J. Effectiveness of Shock-Absorbing Insole for High-Heeled Shoes on Gait: Randomized Controlled Trials. *Healthcare* 2022, *10*.

15. Nigg, B.; Hintzen, S.; Ferber, R. Effect of an Unstable Shoe Construction on Lower Extremity Gait Characteristics. *Clin. Biomech.* **2006**, *21*, 82–88, doi:10.1016/j.clinbiomech.2005.08.013.

16. Stewart, L.; Gibson, J.N.A.; Thomson, C.E. In-Shoe Pressure Distribution in “Unstable” (MBT) Shoes and Flat-Bottomed Training Shoes: A Comparative Study. *Gait Posture* **2007**, *25*, 648–651, doi:https://doi.org/10.1016/j.gaitpost.2006.06.012.

17. Koyama, K.; Naito, H.; Ozaki, H.; Yanagiya, T. Effects of Unstable Shoes on Energy Cost during Treadmill Walking at Various Speeds. *J. Sports Sci. Med.* **2012**, *11*, 632–637.

18. Debbi, E.M.; Wolf, A.; Haim, A. Detecting and Quantifying Global Instability during a Dynamic Task Using Kinetic and Kinematic Gait Parameters. *J. Biomech.* **2012**, *45*, 1366–1371, doi:https://doi.org/10.1016/j.jbiomech.2012.03.007.

19. Buchecker, M.; Stöggl, T.; Müller, E. Spine Kinematics and Trunk Muscle Activity during Bipedal Standing Using Unstable Footwear. *Scand. J. Med. Sci. Sports* **2013**, *23*, e194–e201, doi:10.1111/SMS.12053.

20. Sousa, A.S.P.; Silva, A.; Macedo, R.; Santos, R.; Tavares, J.M.R.S. Influence of Long-Term Wearing of Unstable Shoes on Compensatory Control of Posture: An Electromyography-Based Analysis. *Gait Posture* **2014**, *39*, 98–104.

21. Gu, Y.; Lu, Y.; Mei, Q.; Li, J.; Ren, J. Effects of Different Unstable Sole Construction on Kinematics and Muscle Activity of Lower Limb. *Hum. Mov. Sci.* **2014**, *36*, 46–57, doi:https://doi.org/10.1016/j.humov.2014.04.008.

22. Sousa, A.S.P.; Macedo, R.; Santos, R.; Sousa, F.; Silva, A.; Tavares, J.M.R.S. Influence of Prolonged Wearing of Unstable Shoes on Upright Standing Postural Control. *Hum. Mov. Sci.* **2016**, *45*, 142–153, doi:https://doi.org/10.1016/j.humov.2015.11.015.

23. Salvador-Coloma, P.; Arguisuelas, M.D.; Doménech-Fernández, J.; Sánchez-Zuriaga, D.; Amer-Cuenca, J.J.; Martínez-Gramage, J.; Montañez-Aguilera, F.J.; Lisón, J.F. Effects of Unstable Shoes on Trunk Muscle Activity in Patients with Chronic Low Back Pain. *Gait Posture* **2018**, *64*, 165–168.

24. Khoury-Mireb, M.; Solomonow-Avnon, D.; Rozen, N.; Wolf, A. The Effect of Unstable Shoe Designs on the Variability of Gait Measures. *Gait Posture* **2019**, *69*, 60–65, doi:10.1016/J.GAITPOST.2019.01.017.

25. Zhou, H.; Xu, D.; Quan, W.; Liang, M.; Ugbolue, U.C.; Baker, J.S.; Gu, Y. A Pilot Study of Muscle Force between Normal Shoes and Bionic Shoes during Men Walking and Running Stance Phase Using Opensim. In Proceedings of the Actuators; MDPI, 2021; Vol. 10, p. 274.

26. Zhou, H.; Chen, C.; Xu, D.; Ugbolue, U.C.; Baker, J.S.; Gu, Y. Biomechanical Characteristics between Bionic Shoes and Normal Shoes during the Drop-Landing Phase: A Pilot Study. *Int. J. Environ. Res. Public Health* **2021**, *18*, 3223.

27. Gimunová, M.; Zvonař, M.; Kolářová, K.; Janík, Z.; Mikeska, O.; Musil, R.; Ventruba, P.; Šagat, P. Changes in Lower Extremity Blood Flow during Advancing Phases of Pregnancy and the Effects of Special Footwear. *J. Vasc. Bras.* **2017**, *16*, 214–219.

28. Gimunová, M.; Zvonař, M.; Sebera, M.; Turčínek, P.; Kolářová, K. Special Footwear Designed for Pregnant Women and Its Effect on Kinematic Gait Parameters during Pregnancy and Postpartum Period. *PLoS One* **2020**, *15*, e0232901.

29. Fortenbaugh, D.; Sato, K.; Hitt, J. The Effects of Weightlifting Shoes on Squat Kinematics. In Proceedings of the ISBS-Conference Proceedings Archive; 2010.

30. Sato, K.; Fortenbaugh, D.; Hydock, D.S. Kinematic Changes Using Weightlifting Shoes on Barbell Back Squat. *J. Strength Cond. Res.* **2012**, *26*.

31. Whitting, J.W.; Meir, R.A.; Crowley-McHattan, Z.J.; Holding, R.C. Influence of Footwear Type on Barbell Back Squat Using 50, 70, and 90% of One Repetition Maximum: A Biomechanical Analysis. *J. Strength Cond. Res.* **2016**, *30*, 1085–1092.

32. Legg, H.S.; Glaister, M.; Cleather, D.J.; Goodwin, J.E. The Effect of Weightlifting Shoes on the Kinetics and Kinematics of the Back Squat. *J. Sports Sci.* **2017**, *35*, 508–515, doi:10.1080/02640414.2016.1175652.

33. Clarke, T.E.; Frederick, E.C.; Hamill, C.L. The Effects of Shoe Design Parameters on Rearfoot Control in Running. *Med. Sci. Sports Exerc.* **1983**, *15*, 376–381.

34. Cheung, R.T.H.; Ng, G.Y.F. Efficacy of Motion Control Shoes for Reducing Excessive Rearfoot Motion in Fatigued Runners. *Phys. Ther. Sport* **2007**, *8*, 75–81, doi:https://doi.org/10.1016/j.ptsp.2006.12.002.

35. Rose, A.; Birch, I.; Kuisma, R. Effect of Motion Control Running Shoes Compared with Neutral Shoes on Tibial Rotation during Running. *Physiotherapy* **2011**, *97*, 250–255.

36. Alizadeh, R.; Jafarnezhadgero, A.; Khezri, D. The Acute Effect of Using Anti-Pronation Insoles on the Frequency Content of Lower Limb Muscles in Individuals with Anterior Cruciate Ligament Reconstruction with Pronated Feet during the Stance Phase of Walking. *Iran. J. Rehabil. Res.* **2024**, *10*, 0.

37. Sinclair, J.; Greenhalgh, A.; Edmundson, C.J.; Brooks, D.; Hobbs, S.J. Gender Differences in the Kinetics and Kinematics of Distance Running: Implications for Footwear Design. *Int. J. Sport. Sci. Eng.* **2012**, *6*, 118–128.

38. Chambon, N.; Delattre, N.; Guéguen, N.; Berton, E.; Rao, G. Is Midsole Thickness a Key Parameter for the Running Pattern? *Gait Posture* **2014**, *40*, 58–63.

39. Squadrone, R.; Rodano, R.; Hamill, J.; Preatoni, E. Acute Effect of Different Minimalist Shoes on Foot Strike Pattern and Kinematics in Rearfoot Strikers during Running. *J. Sports Sci.* **2015**, *33*, 1196–1204.

40. Law, M.H.C.; Choi, E.M.F.; Law, S.H.Y.; Chan, S.S.C.; Wong, S.M.S.; Ching, E.C.K.; Chan, Z.Y.S.; Zhang, J.H.; Lam, G.W.K.; Lau, F.O.Y.; et al. Effects of Footwear Midsole Thickness on Running Biomechanics. *J. Sports Sci.* **2019**, *37*, 1004–1010, doi:10.1080/02640414.2018.1538066.

41. Sanno, M.; Epro, G.; Brüggemann, G.-P.; Willwacher, S. Running into Fatigue: The Effects of Footwear on Kinematics, Kinetics, and Energetics. *Med. Sci. Sports Exerc.* **2020**.

42. Dehaghani, M.R.; Nourani, A.; Arjmand, N. Effects of Auxetic Shoe on Lumbar Spine Kinematics and Kinetics during Gait and Drop Vertical Jump by a Combined in Vivo and Modeling Investigation. *Sci. Rep.* **2022**, *12*, 18326.

43. Alonzo, R.; Teo, C.; Pan, J.W.; Teng, P.S.P.; Sterzing, T.; Kong, P.W. Effects of Basketball Shoe Midsole Hardness on Lower Extremity Biomechanics and Perception during Drop Jumping from Different Heights. *Appl. Sci.* **2020**, *10*, 3594, doi:10.3390/app10103594.
